# Shift Towards Syndiniales Prevalence in Protist Communities During a Marine Heatwave

**DOI:** 10.1111/1758-2229.70430

**Published:** 2026-10-11

**Authors:** Hanneloor Heynderickx, Ro Allen, Tina C. Summerfield, Kim Currie, Linn J. Hoffmann

**Affiliations:** ^1^ Department of Botany University of Otago Dunedin New Zealand; ^2^ Marine Observation Centre Flanders Marine Institute Oostende Belgium; ^3^ Earth Sciences New Zealand Dunedin New Zealand

## Abstract

Marine heatwaves (MHWs) are becoming more frequent and severe, yet their impact on natural marine protist communities remains poorly understood. Using 18S rRNA gene amplicon sequencing, we examined the effects of the extreme 2017/2018 Austral summer MHW on protist communities in three water masses off the southeast coast of New Zealand. During the MHW, alpha diversity declined significantly in Subtropical Waters (Shannon: 1.78 ± 0.42, *p* = 0.03) and the Front (Shannon: 3.26 ± 0.19, *p* = 0.05), driven by reduced evenness in Subtropical Waters (Pielou: 0.36 ± 0.06, *p* = 0.03) and by both richness and evenness in the Front. Communities also became more homogeneous, with lower dispersion among samples, and the water mass driven structuring of communities seen in non‐MHW summers disappeared during the MHW. The taxonomic composition shifted substantially: the parasitic dinoflagellate *Syndiniales* became the most dominant group in Subtropical Waters and the Southland Front (up 68 and 30 percentage points, respectively), while Dinophyceae decreased by 47% and 20%, respectively. Since host‐parasitoid interactions play a significant role in carbon and nutrient cycling, the strong increase in relative abundance of *Syndiniales* underscores the need to consider biotic interactions, including host‐parasitoid relationships, when studying MHW impacts on marine protists.

## Graphical abstract

During a marine heatwave, protist diversity decreased and communities became more homogeneous. The parasitic dinoflagellate *Syndiniales* became the most dominant group in Subtropical Waters and the Southland Front. Since host‐parasitoid interactions are important in carbon and nutrient cycling, they should be considered when studying heatwave impacts on protist communities.

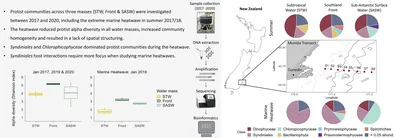

## 1 Introduction

Marine heatwaves (MHWs) are identified as anomalously warm sea‐surface water events that persist for at least 5 days with temperatures exceeding the 90th percentile, based on a 30‐year local baseline climatology (Hobday et al. 2016). They are caused by different localised (e.g., air‐sea heat flux) and regional (e.g., El Niño Southern Oscillation) processes operating across a range of spatial and temporal scales (Hobday et al. 2016). MHWs can result in severe disruption to local ecosystems, such as widespread mortality of foundational species (Thomson et al. 2015; Hughes et al. 2018; Smale et al. 2019; Thomsen et al. 2019; Wernberg et al. 2025; Smith, Burrows, et al. 2023) and benthic invertebrates (Garrabou et al. 2009; Bettim et al. 2025), species distribution shifts (Brodeur et al. 2019; Smith, Free, et al. 2023), and changes in species phenology (Thompson et al. 2022). As a result of climate change, MHWs are predicted to increase in frequency and magnitude (IPCC 2012; Oliver et al. 2019). Hence, the impact of these extreme events on biodiversity patterns, and ecosystem structure and functioning, warrants further research.

The effects of MHWs on marine protist communities are not well studied. However, recent research suggests MHWs can cause changes in protist community composition (Soulié et al. 2022; Ahme et al. 2025; Eglaine et al. 2025). Protists are a diverse group of predominantly unicellular organisms, including various types of phytoplankton as well as heterotrophic flagellates. They dominate the sunlit layer of the ocean and play a key role in the global carbon cycle and biogeochemical fluxes (Falkowski et al. 2008; Laget et al. 2024). They act as producers, consumers, decomposers, and parasites (De Vargas et al. 2015). Their high abundance in the ocean photic zone and their global distribution is attributed to the range of trophic modes that have been characterised within this group, for example, autotrophy, heterotrophy, and mixotrophy (Crane and Grover 2010; Worden et al. 2015).

Parasitic protists such as the dinoflagellate *Syndiniales* are a significant component of marine protist communities (Hatcher and Dunn 2011; Lima‐Mendez et al. 2015; Clarke et al. 2019; Na et al. 2026) and can account for up to 18% of the complex interactions within these systems (Bjorbækmo et al. 2020). *Syndiniales* are opportunistic parasitoids that can infect and kill a wide variety of hosts, including other protists (dinoflagellates, ciliates, and radiolarians) as well as metazoans (Guillou et al. 2008; Anderson and Harvey 2020; Käse et al. 2021). In some cases, they have been shown to cause the decline of toxic microalgal blooms (Chambouvet et al. 2008) and induce mass mortality of fish larvae (Shadrin et al. 2010). *Syndiniales* are highly diverse and are classified into five main groups, with Dino‐Group I and II containing the most diversity (Guillou et al. 2008; Anderson and Harvey 2020; Rizos et al. 2023). Metabarcoding studies to date have mainly encountered members of group II and focused on in vitro studies of the genus *Amoebophrya* (group II) (Chambouvet et al. 2011; Cai et al. 2020; Farhat et al. 2021). Environmental conditions such as temperature, water column depth, mixing, and nutrient concentrations can influence the dynamics of *Syndiniales* populations (Anderson and Harvey 2020), however, we know very little about how climate change might impact them. Further, modelling approaches suggest that the control of dinoflagellate blooms by parasitoids strongly depends on the structure of the plankton community, such as cell densities, grazing on free‐living parasitoid stages, and competition between cells (Alves‐de‐Souza et al. 2015). This lack of general knowledge on abiotic and biotic factors controlling *Syndiniales* abundance and parasitoid–host dynamics in general limits our understanding of fundamental processes that ultimately determine how carbon and nutrients are cycled through the marine food web (Worden et al. 2015; Moran et al. 2022).

The austral summer of 2017/2018 reflected the hottest air/land temperatures New Zealand had encountered in the past 83 years, thereby surpassing the historical record of warmest summer of 1934/1935 (NIWA 2019). This summer coincided with MHW events in the Tasman Sea and New Zealand coastal waters. On the south‐east coast of South Island New Zealand, the MHW persisted for 91 days (Schlegel 2020) and was characterised by an increase of 2°C to 4°C relative to the 1981–2010 average sea surface temperature (NIWA 2019). The intensity of the MHW peaked on January 29, 2018 (Schlegel 2020). On that day, the event was defined as a Category III (severe) MHW according to the categorisation scheme proposed by Hobday et al. (Hobday et al. 2018). Despite growing interest in the biological impacts of MHWs on phytoplankton communities, existing knowledge relies heavily on satellite chlorophyll‐a data (Cavole et al. 2016; Sen Gupta et al. 2020; Chauhan et al. 2023; Zhan et al. 2024), laboratory experiments (Remy et al. 2017), field‐based experiments (Smale et al. 2017), and mesocosm experiments (Rasconi et al. 2017; Soulié et al. 2022; Ahme et al. 2025; Zhang et al. 2025; Eglaine et al. 2025). While mesocosm and experimental studies provide valuable mechanistic insight, they are conducted under controlled or semi controlled conditions and are therefore limited in their ability to capture the full taxonomic breadth and spatial complexity of protist community responses at ecologically realistic scales. In particular, how MHWs restructure natural protist communities in situ, across the environmental heterogeneity of a real ocean frontal system, remains poorly resolved. Through long‐term monitoring of New Zealand waters off the east coast of the South Island, we were able to directly capture the impact of an extreme MHW on photic‐zone protist communities. We identified taxonomic shifts across different water masses over four consecutive summers, thereby addressing the following questions: (i) did the MHW change protist community composition? (ii) did the MHW affect the water mass affiliated protist structuring? and (iii) was species diversity reduced under MHW conditions? To our knowledge, this is the first study to characterise protist community responses to a naturally occurring, unmanipulated MHW event in situ across multiple hydrographically distinct water masses, using a pre‐existing long term monitoring framework rather than an experimental or mesocosm based design.

## 2 Materials & Methods

### 2.1 Site Description

The 65‐km long Munida time‐series transect was established in 1998, based on the transect studied by Jillett (1969), and has been monitored for over two decades mainly to measure changes in ocean acidification. In early autumn 2016, long‐term monitoring of phytoplankton communities was initiated along the transect. The transect itself runs from Taiaroa Head at the tip of the Otago Peninsula (45.770° S 170.720° E) across modified subtropical water (STW) of the Southland Current, followed by the Southland Front (Front) and ends in sub‐Antarctic surface water (SASW) (Jillett 1969; Heath 1981) (Figure 1). The Southland Front is a transition zone between the warm, nutrient‐poor and salty water of STW (15–40 km offshore) and the cold, nutrient‐rich and less saline SASW (> 45 km offshore) (Heath 1981; Hopkins et al. 2010). Moreover, this front corresponds to the Subtropical Convergence but is seen as an independent system due to the interruption of the New Zealand landmass (Heath 1981). It is a site of high variability and complexity regarding its strength, location, width, and nutrient concentrations (Hopkins et al. 2010; Jones et al. 2013). The hydrology and physical oceanography of these water masses were described in earlier research papers (Jillett 1969; Hawke 1989; Currie et al. 2011).

**FIGURE 1:**
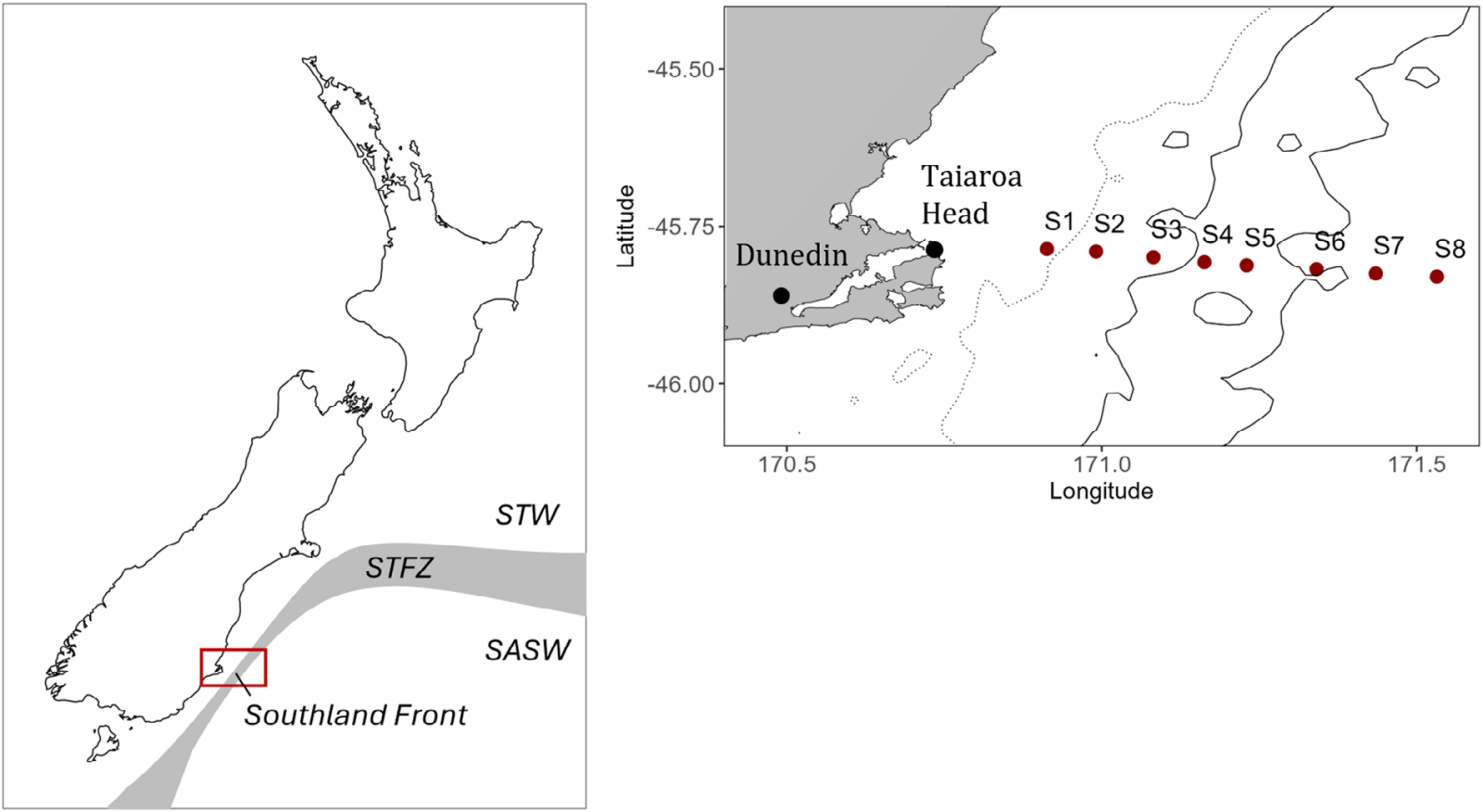
Map of New Zealand illustrating the Subtropical Frontal Zone (STFZ) and the near‐coastal protrusion, the Southland Front, separating Subtropical Water (STW) from sub‐Antarctic Surface Water (SASW). Red boxed area represents the region of the Munida Transect. Red dots present the eight discrete sampling stations (S1–S8) off the southeast coast of South New Zealand.

### 2.2 Sampling

Samples were taken during four single‐day cruises aboard the RV Polaris II between 2017 and 2020 (Heynderickx 2022). Each sampling trip took place in austral summer. Samples were collected at eight discrete stations along the Munida transect (Figure 1). These stations were chosen based on previous research (Currie et al. 2011; Jones et al. 2013; Allen et al. 2020) to allow for an equal representation of the water masses present along the Southland Front. However, since the position of the front is not fixed and shifts between sampling trips, the number of stations falling within each water mass also varied. One seawater sample was collected per station for metabarcoding (2.5 L) at 10 m depth using 12 L Niskin bottles mounted on a CTD Rosette (SBE 19plus V2 SEACAT Profiler CTD). The samples were transferred into autoclaved transparent Nalgene bottles (Thermo Scientific, USA) and subsequently filtered on‐board onto 0.2 μm polyethersulfone membranes (47 mm diameter, Sterlitech) using a closed filtration system. A pressure pump was used and kept at low vacuum (0.2 bar) to prevent rupture from compromising the filter membranes. After filtration, the filters were folded into 1.5 mL microcentrifuge cryo‐vials (National Scientific), snap frozen and stored in liquid nitrogen until they were transferred to −80°C on‐shore awaiting extraction.

### 2.3 Environmental Measurements

Continuous measurements of temperature and salinity were taken from surface waters (2 m depth) for the duration of each single day campaign. Sea‐surface water temperature and conductivity were measured using a SBE45/SBE38 thermosalinograph with a remote temperature sensor (Sea‐Bird Electronics Inc.) and an accuracy of ±0.1°C for sea‐surface temperature. Conductivity, temperature and depth profiles were recorded with CTD casts up to 10 m depth at each sampling station (*n* = 8). Additionally, one seawater sample was collected at each station for the measurement of macronutrients (nitrate, phosphate and silicate) and chlorophyll‐*a* (chl‐*a*) concentrations. Nutrient samples were filtered (Whatman GF/F) on board, collected in HDPE bottles (acid‐washed) and colorimetrically analysed with an Astoria Pacific API 300 micro segmented flow analyser using a digital detector (EPA method 365.5). Water samples for chl‐*a* concentrations were also filtered (Whatman GF/F) on board and frozen in −20°C. Chl‐*a* filters were extracted in 90% acetone for approximately 24 h and measured using a pre‐calibrated Turner Designs Fluorometer as described in Jones et al. (2013). All environmental samples were analysed by NIWA (Table S1).

Temperature (T) and salinity (S) profiles were plotted and overlayed with the discrete temperature and salinity data points from 10 m depth (Figures S1 and S2). The three distinct water masses were identified for each campaign, based on these T/S profiles, on the nutrient concentrations and by following the summer threshold levels for temperature and salinity defined by Jillett (1969). For the MHW event in January 2018, the water masses were identified following the same definitions put forward by Jillett (1969), but with an adjustment of +3.5°C for the temperature threshold. This resulted in the following number of replicates for each water mass: STW non‐MHW *n* = 4, STW MHW *n* = 3, Front non‐MHW *n* = 3, Front MHW *n* = 3, SASW non‐MHW *n* = 13, SASW MHW *n* = 2.

### 2.4 Molecular Methods

#### 2.4.1 DNA Extraction

Frozen filters were thawed and dissected into 2 mm × 1 cm strips using sterilised razor blades. DNA was extracted from the filters with 0.7 M NaCl, 1% Cetyl Trimethylammonium Bromide (CTAB) and 0.1 mm zirconia/silica beads (dnature, BioSpec), following Countway et al. (2005). After the CTAB incubation, sample lysates were further extracted with 1:1 volume of phenol and chloroform. Samples were thoroughly shaken by hand for 30 s and centrifuged (16,000 × g) at room temperature for 5 min. The upper aqueous phase was transferred to a clean 2 mL micro‐centrifuge tube and 1 × volume of chloroform was added. The samples were vortexed for 30 s and centrifuged (16,000 × g) at room temperature for 5 min. DNA was precipitated by adding 1/10 3 M sodium acetate and 2.2 × volume of ice cold 95% ethanol. The solution was vortexed and incubated overnight at −20°C. After centrifugation (20 min at 10,000 × g) at 4°C, DNA precipitate was collected and washed in 250 μL of 70% ethanol followed by a spin (16,000 × g) of 2 min at 4°C to pellet the DNA and subsequently discard the ethanol (the wash step was repeated 3 times). DNA was resuspended in 30 μL of Tris‐EDTA (pH 8) and stored at −80°C. In addition to the frozen samples, sterile clean filters were also processed for DNA extraction to serve as negative lab controls.

#### 2.4.2 18S rRNA Library Preparation

Triplicate PCR mixtures were prepared using the KAPA HiFi HotStart DNA kit (Kapa Biosystems, Boston, MA, USA) for the amplification of the 18S rRNA V4 region (~548 bp). The PCR mixtures were constructed as follows: 5 × KAPA HiFi Buffer (2 mM MgCl_2_), 0.3 mM KAPA dNTP, 0.3 μM forward primer and 0.3 μM reverse primer, 0.5 U KAPA HiFi HotStart DNA Polymerase, and 0.05 mg ml^−1^ Bovine Serum Albumin (BSA, Invitrogen, USA). The forward primer TAReuk454FWD1 (5′–CCAGCASCYGCGGTAATTCC–3′) and reverse primer TAReukREV3 (5′–ACTTTCGTTCTTGATYRA–3′) proposed by Stoeck et al. (2010) were used. These were modified with overhanging tags (forward, 5–ACGACGCTCTTCCGATCT–3′; reverse, 5–CGTGTGCTCTTCCGATC–3′; Griffith et al. 2017) for the addition of Illumina Truseq adaptors and unique dual‐index barcodes during a second PCR round (R2‐PCR). First round amplification (R1‐PCR) was performed using a two‐step PCR programme (adapted from Massana et al. 2015): initial denaturation at 95°C (30 s); followed by step 1–10 cycles at 95°C (30 s), 53°C (30 s), and 72°C (30 s); followed by step 2–15 cycles at 95°C (30 s), 48°C (30 s), and 75°C (30 s); and a final extension period at 72°C (300 s).

Clean‐up of the R1‐PCR products was performed with AMPure XP beads (Beckman Coulter, Brea CA, USA) according to the manufacturer's protocol. Purified products were quantified with a Qubit fluorometer using the Qubit dsDNA High Sensitivity Assay kit (Thermo Fisher Scientific, Waltham, MA, USA) and DNA concentrations were diluted to 1 ng μL^−1^. Thermocycling conditions for the R2‐PCR were as follows: an initial denaturation step at 95°C for 120 s; followed by 10 cycles of 98°C (20 s), 60°C (20 s), and 75°C (20 s); and a final extension period at 72°C (60 s). Purification and quantification of amplicons was done as described for the R1‐PCR. Samples were sequenced using Illumina paired‐end sequencing on an MiSeq platform (Illumina, San Diego, CA, USA) using the V3 (2 × 300 bp) reagent kit at the Otago Genomics Facility (Otago University, New Zealand).

### 2.5 Statistical Analyses

The raw Illumina 18S rRNA sequences were processed in R (R Core Team 2021) following the ‘DADA2’ pipeline to resolve amplicon sequence variants (ASV) (Callahan et al. 2016a, 2016b). Reads were trimmed to remove primers, and low‐quality sequences were removed based on a Q30 score (forward reads at 270 bp and reverse reads at 230 bp). Chimeric ASVs were identified and removed with the r*emoveBimeraDenovo* function. Taxonomy was assigned using *assignTaxonomy* against the Protist Ribosomal Database (v4.14; Guillou et al. 2012), and sequences classified as metazoan were removed prior to further analysis. The dataset was rarefied to an even sequence depth of 14,883 to facilitate comparisons of diversity and community composition among the samples. To confirm this did not bias downstream results, rarefaction was performed 1000 times independently at the selected depth (14,883 reads) and at two additional depths (20,985 and 28,000 reads) spanning the observed range of sequencing depths in the dataset (Figure S3). This repeated‐rarefying approach, recommended by Cameron et al. (2021), characterises the variability introduced by library‐size normalisation. Results were also compared against an alternative, non‐rarified (proportion‐based) normalisation approach (Supporting Information—Section C).

Statistical analyses relied on the ‘phyloseq’ package (McMurdie and Holmes 2013) and the ‘vegan’ package (Oksanen 2020) while all visualisations were done with *ggplot2* (Wickham 2016). Two groups of analysis were used: (I) Beta diversity analyses (PERMANOVA, PERMDISP and dbRDA) to directly test our primary hypothesis that protist community composition differs by water mass and heatwave status and (II) Alpha diversity and SIMPER analyses to provide a complementary and exploratory characterisation of overall diversity patterns and the taxa underlying observed compositional differences. The robustness of the beta‐diversity analyses was additionally assessed, alongside the rarefaction and normalisation checks described above, using two further dissimilarity metrics (Hellinger‐Euclidian and Jaccard), jackknife resampling, and cross‐validation of environmental variable selection in the dbRDA (Supporting Information—Section C).

The MHW event was identified using the heatwave R package implemented in the Marine Heatwave Tracker app (Schlegel 2020). The app uses the daily Optimally Interpolated Sea Surface Temperature OISST; (Reynolds et al. 2007) that is made available by the National Oceanic and Atmospheric Administration (NOAA). Specifically, the AVHRR‐only v2.1 data (Banzon et al. 2020) were used to identify the MHW in austral summer 2017/2018.

Protist alpha diversity (Shannon index, Hill number *q* = 0 (species richness) and Pielou's evenness) was calculated for each sample using *estimate_richness* (“phyloseq”) and compared across water masses and heatwave status. Hill number of order *q* = 0 is equivalent to observed richness, Pielou's evenness quantifies how evenly reads are distributed among taxa, while Shannon captures both richness and evenness. Significance was assessed using the non‐parametric Kruskal‐Wallis test and the post hoc Dunn's test with a Benjamini‐Hochberg correction for false discovery rate.

To test whether community composition differed by water mass and heatwave status, a two‐way Permutational Multivariate Analysis of Variance (PERMANOVA; Anderson 2017; *adonis2* function; ‘vegan’) was performed on Bray‐Curtis dissimilarities. As PERMANOVA can be sensitive to differences in within‐group variation (dispersion), multivariate homogeneity of dispersion was additionally tested using PERMDISP (*Betadisper* function; ‘vegan’) (Anderson 2006) to confirm that significant PERMANOVA results reflected genuine compositional differences rather than unequal within‐group variability. Community patterns were visualised in an ordination plot using unconstrained non‐metric multidimensional distance scaling (nMDS). All beta diversity analyses were performed with 10,000 permutations and a fixed seed of 711 for reproducibility.

The effect of environmental variables (temperature, salinity, phosphate, nitrate, silicate, CO_2_, chlorophyll‐*a* as a proxy for phytoplankton biomass, and pH) on community composition was tested with a distance‐based redundancy analysis (dbRDA; Legendre and Anderson 1999) using Bray‐Curtis dissimilarities. This complements the PERMANOVA test by identifying which specific environmental gradients underlie the compositional differences it detects. As many environmental variables are correlated with one another, candidate variables were tested for co‐linearity by calculating their variance inflation factors (*vif.cca* function; scores > 10 were removed). Following identification of co‐linearity, remaining variables were fitted in the model through forward selection (*ordiR2step* function; ‘vegan’). The significance of the overall constrained ordination model and individual explanatory variables was assessed using permutation‐based ANOVA (*anova.cca*, ‘vegan’).

To identify which taxa (at class and ASV level) contributed to compositional differences between summer and MHW conditions within each water mass, the similarity percentage (SIMPER; Clarke 1993) analysis was used on relative abundance data using Bray‐Curtis dissimilarities and 10,000 permutations (*Simper* function; ‘vegan’). SIMPER identifies taxa that vary most between groups rather than taxa that are strictly indicative of a given group (Warton et al. 2012). Finally, differences in nutrient concentrations between summer and MHW conditions were tested using a one‐sided Wilcoxon test.

## 3 Results

### 3.1 Hydrographic Parameters

During the MHW event, the continuous sea surface temperatures (2 m) ranged from 17.1°C to 18.4°C, while the temperatures at 10 m depth measured through the CTD cast ranged from 14.7°C to 16.7°C (Heynderickx 2022). The mean seawater temperatures were significantly higher during the MHW across the different water masses (Wilcoxon; *p* < 0.05) compared to the mean seawater temperatures collected during the summer campaigns in 2017, 2019, and 2020 (Table 1). Sea surface salinity (SSS), however, did not significantly differ during the MHW from the non‐MHW summers for any of the three water masses. In general, SSS showed a strong spatial structure, with the highest salinity values observed for STW (34.8‰, at 2 and 10 m) and the lowest values for SASW (34.3‰ at 2 and 10 m; Table 1). The same SSS signals persisted during the MHW.

**TABLE 1:** Mean values for sea surface temperature and salinity (± standard deviation) for the summer cruises including a MHW event. SST: Sea Surface Temperature (°C) and SSS: Seas Surface Salinity (‰) values. The SST and SSS values are given at two different sampling depths (2 m and 10 m). The MHW event represents the 2018 January cruise and is compared to the non‐MHW cruises from January 2017, 2019, and 2020. The water masses are as follows: Subtropical Water (STW), Southland Front (Front), and sub‐Antarctic Surface Water (SASW).

	Non‐MHW summers				MHW event			
	Surface 2 m		Depth 10 m		Surface 2 m		Depth 10 m	
	SST	SSS	SST	SSS	SST	SSS	SST	SSS
STW	13.8 ± 0.5	34.8 ± 0.0	13.1 ± 0.5	34.8 ± 0.1	17.8 ± 0.1	34.8 ± 0.0	16.3 ± 0.3	34.8 ± 0.0
Front	12.7 ± 0.3	34.6 ± 0.1	11.8 ± 0.5	34.5 ± 0.1	17.4 ± 0.0	34.8 ± 0.0	15.3 ± 0.5	34.7 ± 0.1
SASW	12.1 ± 0.6	34.3 ± 0.1	11.6 ± 0.6	34.3 ± 0.1	17.3 ± 0.2	34.3 ± 0.0	15.2 ± 0.1	34.4 ± 0.0
Min	11.3	33.3	10.9	34.2	17.1	34.3	14.7	34.3
Max	15.9	34.8	14.8	34.8	18.4	34.8	16.7	34.9

### 3.2 Macronutrient Concentrations and Chlorophyll‐*a*

In SASW, both nitrate and phosphate concentrations were significantly lower during the MHW event (nitrate 6.51 ± 0.09 μM, phosphate 0.44 ± 0.01 μM; one‐sided Wilcoxon; *p*‐values = 0.01 for both; Table 2) compared to the other non‐MHW summers (nitrate 8.83 ± 0.54 μM, phosphate 0.67 ± 0.04 μM; January 2017, 2019 and 2020). In STW and the Front, no significant differences were detected for these macronutrients. Nitrate and phosphate continued to display a consistent spatial pattern during the MHW with lower values in the coastal adjacent STW and peak concentrations for the offshore waters (Front & SASW) (Table 2 and Table S1). No differences in silicate values were detected in any of the water masses during the MHW event.

**TABLE 2:** Mean macronutrient and chlorophyll‐*a* concentrations (± standard deviation) for each individual water mass. ‘Non‐MHW’ represents the summer cruises in January 2017, 2019, and 2020. MHW represents the January 2018 cruise. Water masses: Subtropical Water (STW), Southland Front (Front), and sub‐Antarctic Surface Water (SASW). Highlighted cells with an asterisk (*): Significant difference *p* < 0.05, one‐sided Wilcoxon test between MHW and non‐MHW values.

		STW	Front	SASW
Nitrate	Non‐MHW	1.70 ± 0.61	5.10 ± 4.24	8.83 ± 0.54
(μM)	MHW	1.21 ± 0.67	5.71 ± 0.25	6.51 ± 0.09*
Phosphate	Non‐MHW	0.28 ± 0.09	0.47 ± 0.28	0.67 ± 0.04
(μM)	MHW	0.16 ± 0.06	0.41 ± 0.01	0.44 ± 0.01*
Silicate	Non‐MHW	1.11 ± 0.23	1.16 ± 0.44	0.53 ± 0.14
(μM)	MHW	0.96 ± 0.22	0.58 ± 0.06	0.44 ± 0.01
Chl‐*a*	Non‐MHW	1.41 ± 0.12	1.18 ± 0.83	0.29 ± 0.17
(mg m^−3^)	MHW	0.27 ± 0.02*	0.21 ± 0.01	0.20 ± 0.04

Chl‐*a* concentrations ranged from 0.14–2.06 mg m^−3^, with the lowest values found in SASW and the highest in STW (Table S1). Chl‐*a* values exceeding the 1.5 mg m^−3^ bloom indicator threshold were detected in STW for the January 2017 samples and in the Front for the January 2019 samples. During the MHW, chl‐*a* concentrations were consistent across all water masses and did not exceed 0.29 mg m^−3^. For STW, chl‐*a* was found to be significantly lower during the MHW (1.41 ± 0.12 mg m^−3^ non‐MHW vs. 0.27 ± 0.02 mg m^−3^ MHW; one‐sided Wilcoxon; *p* = 0.03; Table 2).

### 3.3 Decreased Alpha Diversity in STW and Front During the MHW

Protist alpha diversity measures were significantly lower for STW (Shannon: 1.78 ± 0.42 during the MHW; X^2^ = 4.5, df = 1, *p* = 0.03) and the Front (Shannon: 3.26 ± 0.19 during the MHW; X^2^ = 3.9, df = 1, *p* = 0.05) compared to the other summer months (Figure 2a; full group values are given in Table S2). In STW, the significantly lower Pielou value (0.36 ± 0.06 during the MHW; X^2^ = 4.5, df = 1, *p* = 0.03) was the driver behind the difference in alpha diversity (Figure 2c), while richness (Hill number *q* = 0) did not differ significantly (X^2^ = 2.0, df = 1, *p* = 0.16). The reduced protist alpha diversity in the Front was a combination of variation in both richness and evenness (Hill number *q* = 0 and Pielou; X^2^ = 3.9, df = 1, *p* = 0.05; Figure 2b,c). No significant differences were observed for the SASW communities during the MHW (*n* = 2), likely reflecting this reduced sample size as well as interannual variability in diversity measures among the 2017/2019/2020 summers. Differences across the water masses were observed during the 2017/2019/2020 summers (Hill number q = 0: STW 217.8 ± 57.2 vs. Front 499.0 ± 11.8; X^2^ = 7.18, df = 2, *p* = 0.03) between STW and the Front (Hill number q = 0; Z = 2.66, df = 1, *p* = 0.02), as well as during the MHW event (Shannon; X^2^ = 6.25, df = 2, *p* = 0.04) between STW and the Front (Shannon; Z = 2.50, df = 1, *p* = 0.04), comparing the same STW and Front MHW values reported above.

**FIGURE 2:**
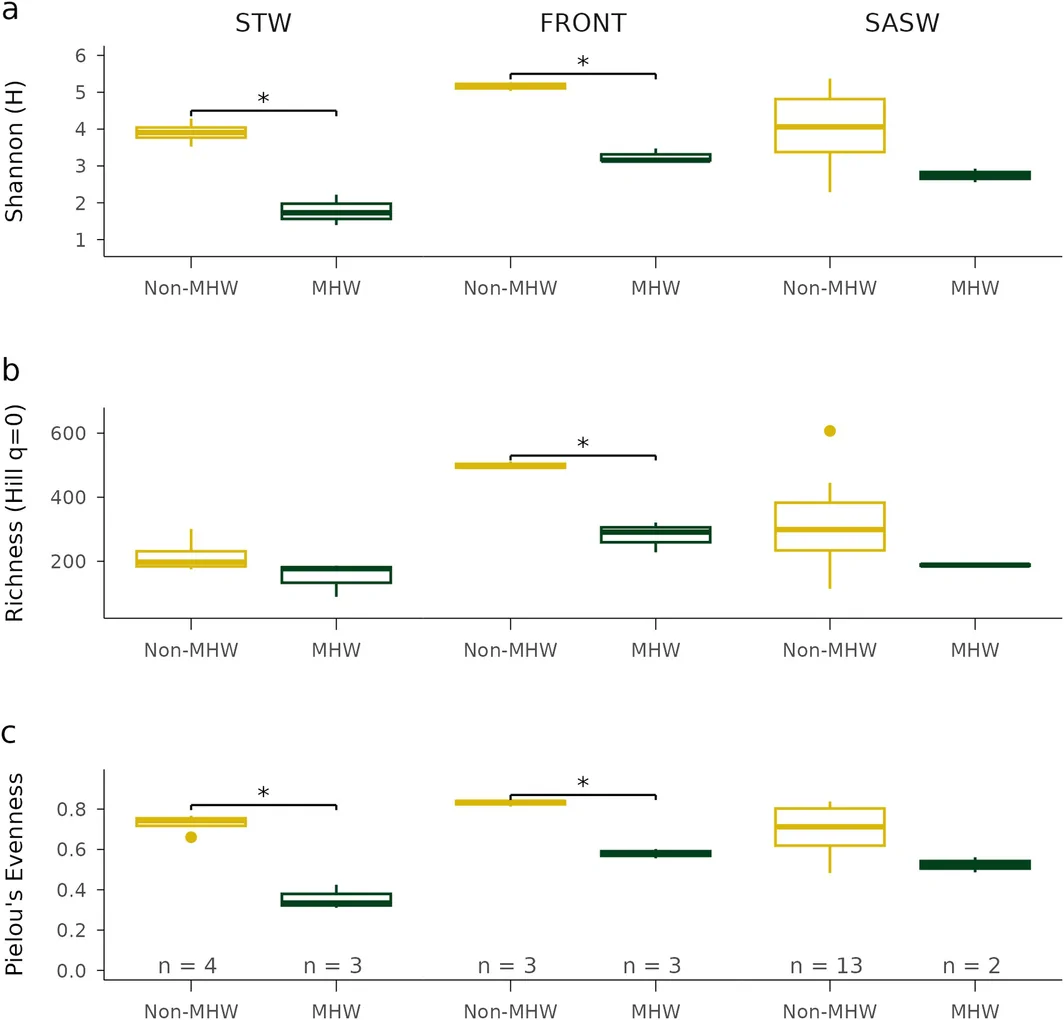
Comparison of protist alpha diversity indices for each water mass during the marine heatwave. Non‐MHW: January 2017, 2019 and 2020 summer cruises, MHW: Marine heatwave event (January 2018 cruise). Sample sizes (*n*) per group are displayed and are the same for each diversity index. Water masses: Subtropical Water (STW), Southland Front (FRONT), and sub‐Antarctic Surface Water (SASW). Significance level at *p* < 0.05 (*).

### 3.4 No Community Structure Detected Across Water Masses During the MHW

PERMANOVA analysis showed that both water mass (R^2^ = 18.0%, pseudo‐*F*_2,22_ = 3.70, *p* < 0.001) and the MHW event (R^2^ = 18.4%, pseudo‐*F*_1,22_ = 7.58, *p* < 0.001) affected protist community composition, and that there was a significant interaction between these factors (R^2^ = 10.1%, pseudo‐*F*_2,22_ = 2.08, *p* < 0.01) (Figure 3a). The assumption of homogeneity of variance among groups was violated for the community comparisons between samples from the MHW cruise and samples from the non‐MHW cruises, with greater average dispersion (mean distance to centroid: 0.52 vs. 0.37) observed among the pooled non‐MHW samples than among the MHW samples (PERMDISP; F_1,26_ = 10.5, *p* = 0.003). Despite the variance in dispersions, separate protist community clusters can be distinguished for the MHW on the nMDS ordination (Figure 3a). Additionally, there appears to be less variance in community composition within each water mass during the MHW (Figure 3a). Pairwise analysis further revealed that the protist communities sampled in the non‐MHW summers were structured according to the water masses (STW‐Front: p.adj = 0.041; STW‐SASW: p.adj = 0.006; Front‐SASW: p.adj = 0.045; PERMDISP: F_2,17_ = 1.77, *p* = 0.2), while this was not observed during the MHW event (STW‐Front, STW‐SASW, Front‐SASW: all *p*‐values = 0.1, the minimum achievable value given the small sample sizes within this single cruise; PERMDISP: F_2,5_ = 0.62, *p* = 0.58).

**FIGURE 3:**
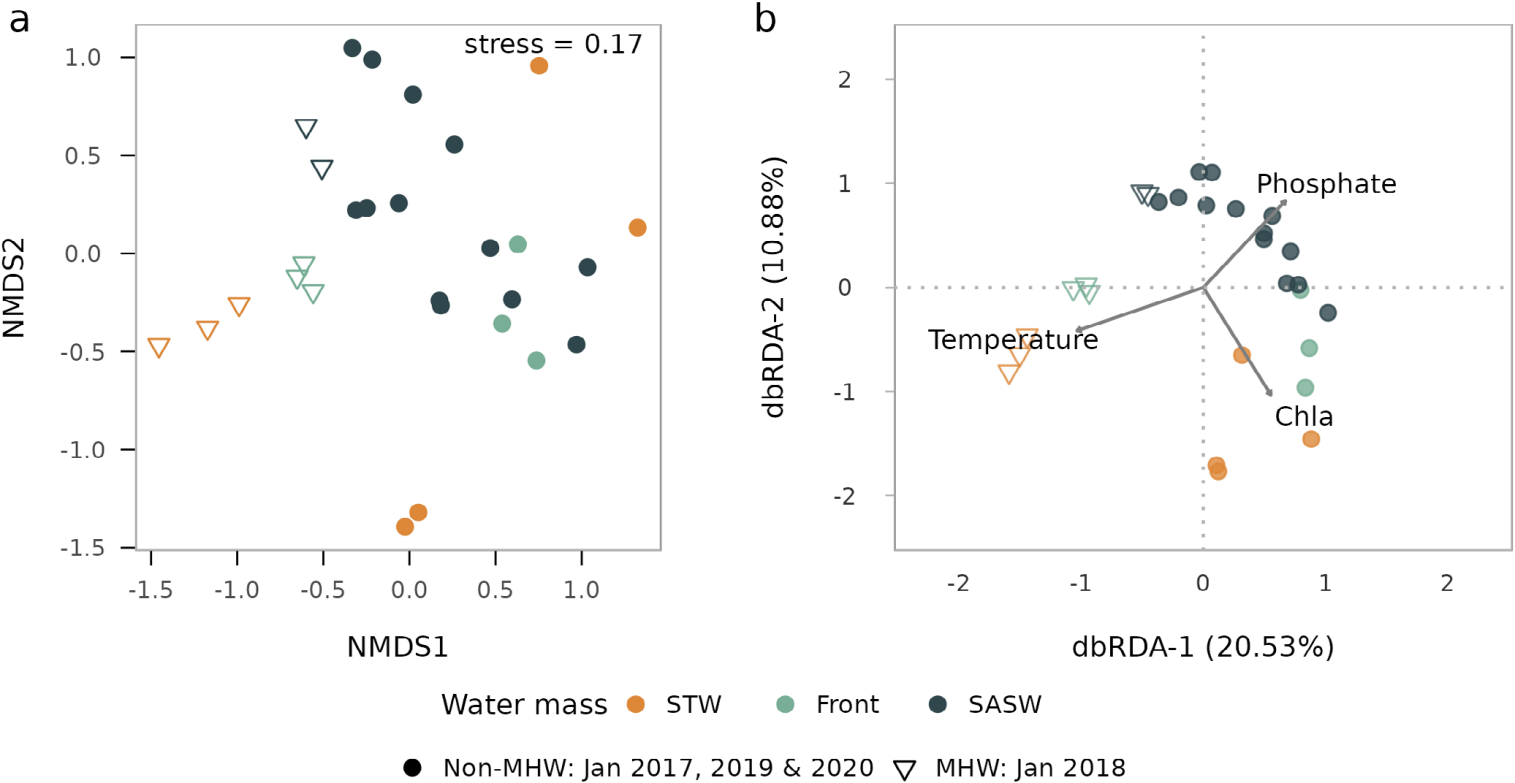
Protist community compositions. (a) Unconstrained non‐metric multidimensional scaling (nMDS). (b) Distance‐based redundancy analysis (dbRDA) of the protist communities. Temperature, chlorophyll‐*a* (chla) and phosphate & nitrate were the defining variables of the community variation. The constrained variables accounted for 38.4% of the total variation in protist communities and were all found to be significant (*p* < 0.05). Colour shows the different water masses: Subtropical Water (STW), Southland Front (Front) and sub‐Antarctic Surface Water (SASW). Shapes show the non‐MHW samples collected during the summer cruises in January 2017, 2019, and 2020 versus MHW samples collected in January 2018.

To quantify the explanatory power of the environmental variables on the structure of the protist communities, distance‐based RDA (dbRDA) analysis was used. From the eight candidate variables, nitrate was excluded due to high collinearity with temperature and phosphate (VIF = 22.8). Next, forward selection identified temperature, chl‐*a*, and phosphate (adjusted R^2^ = 0.15, 0.25, and 0.31 respectively and cumulative) for the final minimal model (Figure 3b). This model constrained 38.4% of the total variation measured in the community composition (unadjusted R^2^). The strongest individual predictor was temperature (R^2^ = 18.0% of total variation). The first constrained ordination axis (CAP1), along which temperature loaded most strongly, explained 20.5% of the total community variation, clustering the MHW protist communities affiliated with STW and the Front separately from all other samples, including the MHW—SASW samples (Figure 3b). The global dbRDA model and each individual explanatory variable were all significant (*p* < 0.05).

To assess the robustness of these findings, we conducted a series of sensitivity analyses examining the influence of rarefaction depth and normalisation approach, dissimilarity metric, individual sample influence, and variable selection procedure on the primary PERMANOVA and dbRDA results (Supporting Information, Section C). The heatwave—water mass interaction remained statistically significant across all conditions tested, including 1000 independent rarefaction draws at three candidate depths (14,883; 20,985 and 28,000 reads) (Table S3 and Figure S3), an alternative non‐rarefied (proportion based) normalisation (Table S4), three dissimilarity metrics (Bray‐Curtis, Hellinger‐Euclidean and Jaccard) (Table S5), and the removal of any single sample (Table S6). Environmental variables selected through forward selection in the dbRDA analysis (temperature, chl‐*a* and phosphate) were retained in 100% jackknife (leave‐one‐sample‐out) iterations and were not excluded under backward elimination, providing convergent support for their identification as primary drivers of community composition (Table S7). A more detailed description of these validation analyses is presented in the Supporting Information—Section C.

### 3.5 Taxonomic Community Composition

The changes in taxonomic composition associated with the MHW were mainly defined by both large increases and reductions in relative abundance of different *Dinoflagellata* taxa. Most notably during the MHW, relative abundance of *Syndiniales* increased from 12.9% to 81.0% in STW and 18.3% to 48.4% in the Front and remained unchanged at 23% in SASW (Table 3). In addition, there were increases in *Chlorophyta* (from 6.5% to 15.3% in the Front and 16.6% to 52.0% in SASW) and decreases in diatoms (from 13% to 0.25% in STW and 9.64% to 1.80% in the Front) (Table 3 and Figure S4). Additionally, changes in less abundant groups were also noticed for *Haptophyta* and heterotrophs *Ciliophora*, *Radiolaria*, and *Cercozoa* (Figure S4). For comparisons with the MHW event, the mean relative abundance of protist communities from the 2017, 2019, and 2020 summers were aggregated per water mass (Table 3).

**TABLE 3:** Relative abundance of the top three protist divisions and affiliated classes ± standard error. ‘Non‐MHW’ represents the means of summer cruises in January 2017, 2019, and 2020. MHW represents the January 2018 cruise. STW: Subtropical water, Front: Southland front, SASW: Sub‐Antarctic surface water.

Division	Class	STW		Front		SASW	
		Non‐MHW	MHW	Non‐MWH	MHW	Non‐MWH	MHW
Dinoflagellata		64.5 (±0.26)	85.4 (±0.08)	51.5 (±0.06)	61.4 (±0.05)	56.6 (±0.07)	40.0 (±0.10)
	Syndiniales*	12.9 (± 0.22)	81.0 (±0.09)	18.3 (±0.04)	48.4 (±0.04)	23.0 (±0.05)	23.0 (±0.07)
	Dinophyceae	51.4 (± 0.28)	4.40 (±0.06)	33.1 (±0.09)	12.9 (±0.06)	33.4 (±0.10)	17.0 (±0.18)
Chlorophyta		5.07 (±0.07)	5.90 (±0.19)	6.52 (±0.08)	15.3 (±0.10)	16.6 (±0.22)	52.0 (±0.40)
	Chloropicophyceae	1.20 (±0.03)	2.10 (±0.20)	1.53 (±0.20)	11.4 (±0.10)	14.2 (±0.45)	51.0 (±0.60)
	Mamiellophyceae	1.98 (±0.02)	0.49 (±0.08)	4.62 (±0.06)	3.80 (±0.18)	2.26 (±0.16)	0.19 (±0.09)
	Pyramimonadophyceae	1.40 (±0.07)	0.04 (±0.00)	0.34 (±0.05)	0.004 (±0.00)	0.04 (±0.006)	0.01 (±0.00)
	Trebouxophyceae	0.35 (±0.03)	3.10 (±0.60)	0.02 (±0.01)	0.04 (±0.02)	0.001 (±0.00)	0.00 (±0.00)
Ochrophyta		13.7 (±0.11)	1.00 (±0.03)	12.6 (±0.14)	4.70 (±0.05)	6.18 (±0.04)	0.30 (±0.02)
	Bacillariophyta	13.0 (±0.10)	0.25 (±0.02)	9.64 (±0.19)	1.80 (±0.04)	2.60 (±0.05)	0.13 (±0.03)
	Pelagophyceae	0.31 (±0.32)	0.30 (±0.03)	1.54 (±0.19)	2.50 (±0.10)	2.86 (±0.07)	0.06 (±0.00)
	Dictyochophyceae	0.21 (±0.02)	0.30 (±0.03)	0.86 (±0.05)	0.20 (±0.01)	0.35 (±0.02)	0.08 (±0.01)

* *Syndiniales* is classified as a Class rank following the Protist Ribosomal Database (v4.14).

The dinoflagellates *Syndiniales* and *Dinophyceae* were identified as the major classes that significantly contributed to the dissimilarity between the summer and MHW protist communities in STW (SIMPER; cumulative contribution of 73%, *p* = 0.001 and 0.037 respectively). In the Front, the SIMPER analysis identified *Syndiniales* (*p* = 0.001), *Dinophyceae* (*p* = 0.001), *Chloropicophyceae* (*p* = 0.001), and *Bacillariophyta* (*p* = 0.101) to have contributed the most to this dissimilarity (cumulative contribution of 72%).

During the MHW, *Syndiniales* dominated the relative abundance of the protist communities with 81% in STW and 48.4% in the Front (Table 3 and Figure S4). To examine the diversity within *Syndiniales*, the relative abundance of this parasitic protist was visualised at a higher taxonomic resolution and showed that sequences primarily belonged to Dino‐Goup‐I and to a lesser extent Dino‐Group‐II, in all water masses (Figure 4). The increase in *Syndiniales* detected during the MHW was driven by the high relative abundance of a single amplicon sequence variant (ASV_1), identified as *Dino‐Group‐I‐Clade‐1* (ASV level: 68.1% in STW; 39.7% in the Front; Figures S5 and S6). The major contribution of *Dinophyceae* to the dissimilarity in protist communities, in both STW and the Front, was characterised by a decline in multiple *Dinophyceae* genera, including *Gyrodinium*, *Gymnodinium*, *Gonyaulax* and *Tripos* (Figures S5 and S6).

**FIGURE 4:**
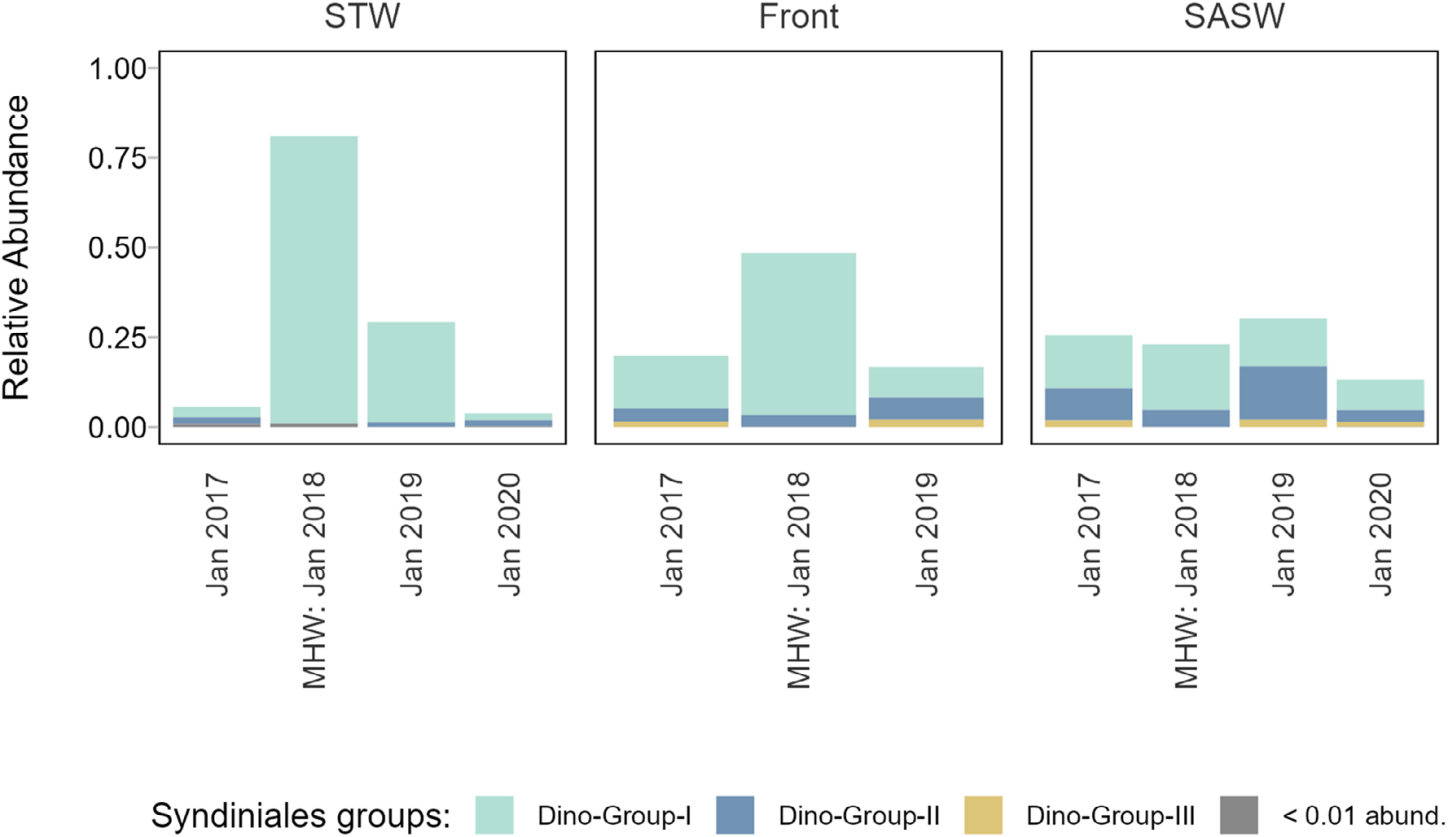
Relative abundances of *Syndiniales* groups during the different summers and across the three water masses. MHW: Marine heatwave, STW: Subtropical water, Front: Southland front, SASW: Sub‐Antarctic surface water.

In SASW, the two most contributing classes were *Chloropicophyceae* (*p* = 0.008) and *Dinophyceae* (*p* = 0.020) with a cumulative contribution of 59% (SIMPER), while the relative abundance of *Syndiniales* did not change (Table 3). A large increase in *Chloropicophyceae* was observed during the MHW due to the positive shift of one species, *Chloroparvula pacifica* (Figure S7). This change was also noticed in the Front (Figure S6). Lastly, *Bacillariophyta* (diatoms; *Ochrophyta*) and *Spirotrichea* (ciliates; *Ciliophora*) relative abundances were reduced during the MHW in all water masses; however, the contribution of diatoms and ciliates to the dissimilarity between summer and MHW communities was only significant in STW (SIMPER; *p* = 0.001 and *p* = 0.029, respectively).

It should be noted that dinoflagellates have large genomes and high rRNA gene copy numbers, while diatoms generally have much lower copy numbers. The lower apparent abundance of diatoms in our rDNA dataset should therefore be interpreted with caution and may underrepresent their actual cell abundance in the community. We therefore compared the relative abundance of ASVs within the dinoflagellate community only to reduce the impact of genome size (Figure 5). Considering only the dinoflagellate ASVs, there is an increase in the relative abundance of the *Syndiniales* during the marine MHW in January 2018, in samples from the STW and the front but to a much lesser extent in the SASW.

**FIGURE 5:**
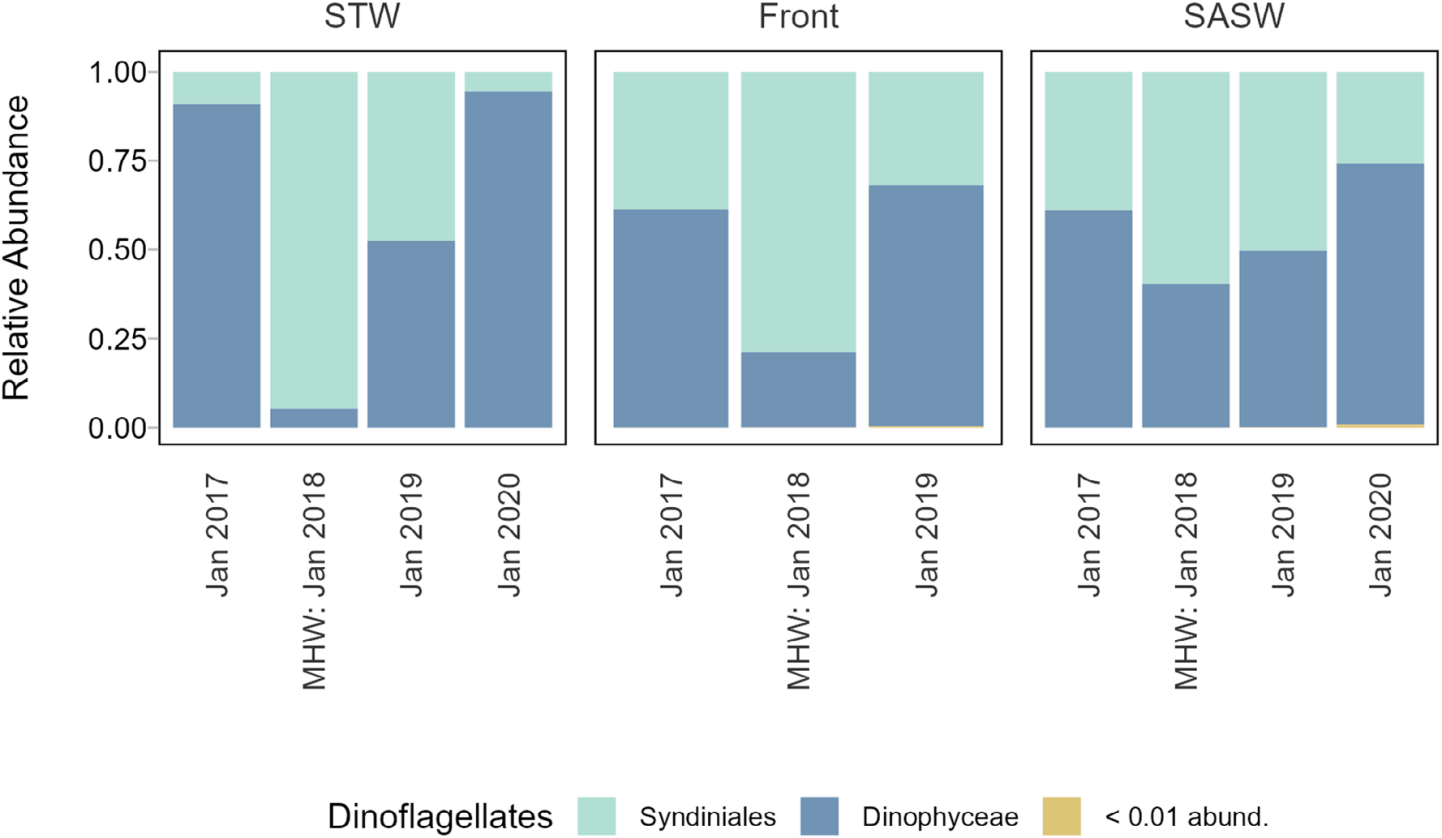
Relative abundance of dinoflagellate ASVs in water samples at different time points from the Sub tropical water (STW), Front and sub‐Antarctic surface water (SASW).

## 4 Discussion

### 4.1 Reduced Alpha and Beta Diversity

By capturing an extreme, unmanipulated MHW event through an existing long term monitoring programme, this study provides the first in situ characterisation of how natural protist communities across a dynamic frontal system respond to marine heatwave conditions, revealing both a loss of spatial community structuring and a marked shift in taxonomic dominance towards parasitic *Syndiniales*. Previous research shows that protist communities are highly structured across the Southland Front off the southeast coast of South New Zealand, with distinct communities in STW, the Front, and SASW (Allen et al. 2020). The same structuring was present for the 2017, 2019 and 2020 summer cruises. During the extreme MHW of Austral summer 2017/2018, the protist communities were more homogenous within each water mass and did not demonstrate spatial structuring across the different water masses. It has been suggested that more homogenous communities under MHW conditions are caused by a facilitation of stress resistant species while more vulnerable species decrease (Scheibling and Gagnon 2009; Castro et al. 2021). Similar observations have been made in communities under ocean acidification conditions (Allen et al. 2021). Congruent with previous studies (Rasconi et al. 2017; Liu et al. 2022), the protist alpha diversity was reduced in all three water masses (STW, Front and SASW) during the MHW even though this reduction was not significant for SASW (Figure 2). Increased sea surface temperatures, as experienced during the MHW, have been shown to enhance thermal stratification and shoaling of the mixed layer (Sarmiento et al. 2004; Behrenfeld et al. 2006). These processes would prevent the vertical nutrient exchange required to sustain high productivity and abundance in the euphotic zone and could result in a significant reduction of species diversity. A community dominated by fewer, more tolerant taxa is likely to offer a narrower range of food resources and altered energy pathways to higher trophic levels, potentially reducing the resilience of the food web to further environmental change. Additionally, by looking at species evenness (Figure 2) and the ASV heatmaps (Figures S5–S7) it is apparent that during the MHW, protist communities in all three water masses became dominated by a small number of taxa already present during the summer, rather than new species being introduced under the MHW conditions.

### 4.2 Shifts in Taxonomic Composition of Protist Communities

The change in community composition associated with the MHW event was defined by a shift in dominance from *Dinophyceae* to *Syndiniales*, in both STW and the Front (Figure S4). This shift is ecologically significant, as *Syndiniales* exert strong top‐down control on other protists and metazoans and contribute to the labile dissolved organic carbon pool through host infection, thereby reducing the transfer of energy to higher trophic levels (Lima‐Mendez et al. 2015; Moran et al. 2022). A rise in their dominance during the MHW therefore points to a potential shift in how carbon and energy moved through the food web in STW and the Front during this event. However, since these results describe protist community responses along a single frontal transect during one MHW event, the results should be interpreted as a case study rather than a general response of Southern Ocean or subtropical frontal systems to MHW conditions.

Here, temperature was found to be the strongest environmental factor responsible for the protist community variability in STW and the Front during the MHW (dbRDA analysis). The positive correlation between *Syndiniales* abundance and sea surface temperature has been previously observed in short‐ and long‐term temporal studies on coastal waters, the open ocean and polar systems (Lima‐Mendez et al. 2015; Berdjeb et al. 2018; Anderson and Harvey 2020). In SASW, the change in community composition was defined by a large increase of *Chloropicophyceae*. Although temperature changes in SASW were of the same order as in STW and Front during the MHW, the relative abundance of *Syndiniales* in this water mass remained completely unchanged. Possibly, changes in the species composition of the host community of *Syndiniales* during MHWs are a stronger driver for *Syndiniales* abundance than temperature alone. The same observation was made in previous studies, where variables such as biotic interactions were argued to be a major predictor in *Syndiniales* community structuring (Lima‐Mendez et al. 2015; Anderson and Harvey 2020). While this study did not formally analyse community interactions, some interesting patterns among the taxonomic groups were observed.

A limitation of the 18S rRNA amplicon sequencing approach is variation in eukaryotic ribosomal RNA gene copy number (Gong and Marchetti 2019). We have detected a substantial change in the community, although the precise relation between relative abundance and environmental abundance is not clear (Nagarkar and Palenik 2023). We have focused on changes to abundant taxa as the uneven sample sizes across certain time points in some water masses limit our statistical power to detect variation in rare taxa. While changes in relative abundance of the native community during a marine heat wave provide new insight into correlations with environmental parameters, it does preclude definitive causal conclusions.

With the positive shift in abundance of *Syndiniales* in STW and the Front, a large reduction in *Dinophyceae* was observed, as well as a decrease in diatoms, ciliates, haptophytes, and an increase in *Chloropicophyceae* (Figure S4). Most *Syndiniales* host‐interactions identified to date have involved positive relationships with *Dinophyceae* and ciliates, while diatoms have primarily been associated with negative interactions (Lima‐Mendez et al. 2015; Anderson and Harvey 2020; Käse et al. 2021). These co‐occurring shifts raise the possibility that *Dinophyceae*, diatoms, and *Syndiniales* are connected through host‐parasitoid interactions in our system. Even though ciliates were observed in our study, none of them were classified as tintinnids, which are recognised as common hosts or predators of certain *Syndiniales* Dino‐Group‐I members (Bachvaroff et al. 2012; Lima‐Mendez et al. 2015). The most abundant *Dinophyceae* ASVs across all water masses belonged to the genera *Gyrodinium* and *Gymnodinium*, both known hosts of *Syndiniales* (Anderson and Harvey 2020). Moreover, *Dinophyceae* were present in relative equal abundances to *Syndiniales* during the non‐MHW summers and were mostly represented by the same few highly abundant ASVs in all three water masses (Figures S5–S7). For diatoms, most interactions identified in the past have been negative (Lima‐Mendez et al. 2015; Anderson and Harvey 2020), however, Sassenhagen et al. (2020) did discuss potential *Syndiniales* Dino‐Group I infections in *Thalassiosira*, *Eucampia*, and *Corethron*, while Anderson and Harvey (2020) found both a negative (Dino‐Group‐III and *Thalassiosira minuscula*) and positive link (Dino‐Group‐I and *Cyclotella* sp.) with diatoms. In our study, the diatom *Thalassiosira delicatula* was found among the top most abundant ASVs in both STW and the Front during the non‐MHW summers. Both *Dinophyceae* and diatom groups mentioned here could potentially be linked to *Syndiniales* in STW and the Front based on the interactions found in earlier studies and their co‐occurrence in these water masses. In SASW, the same reasoning would hold true for *Dinophyceae* but not for diatoms as this taxonomic group was virtually absent in this water mass. Although it would not be uncommon to find different *Syndiniales*‐host interactions across the transect as community structuring was shown to be water mass specific for the non‐MHW summer, thereby providing a different pool of potential host organisms for the parasitoid.

The high relative abundance of *Syndiniales* alongside a sharp decline of *Dinophyceae* and diatoms during the MHW in STW and the Front is consistent with an extensive infection by these parasitoids followed by a crash of the potential hosts and release of free‐living dinospores into the environment (Coats and Park 2002; Guillou et al. 2008; Anderson and Harvey 2020). Since metabarcoding does not distinguish infection stage from free living dinospores and no direct host‐parasitoid interaction data were collected in this study this interpretation remains speculative at this stage. However, it is known that *Syndiniales* compared to *Dinophyceae* have low chromosome numbers (Loeblich 1976), and therefore their high relative abundance observed during the MHW event is likely to reflect increased biomass.

Different dynamics were at play in SASW, where a decline in *Dinophyceae* populations was not accompanied by a rise in *Syndiniales* abundance. The most significant change in this water mass appeared within *Chloropicophyceae*, specifically by *Chloroparvula pacifica*. It should be noted that this pattern is based on only two samples collected in SASW during the MHW and should therefore be interpreted with caution. The suspected events in STW and the Front during the MHW likely happened following an initial bloom event where the host populations would have essentially gone through a similar increase in biomass before experiencing a crash. SASW is characterised as a high‐nutrient low‐chlorophyll water mass where phytoplankton productivity is limited by iron availability (Banse 1996; Moore et al. 2001), making it unlikely that a bloom event took place here. One possible explanation is that without an increase in host populations, *Syndiniales* infections would have retained more stable levels. Concerning *Chloropicophyceae* members, so far no positive or negative associations with *Syndiniales* have been described. Due to a lack of direct evidence of host‐parasitoid interactions in this study and a scarcity of literature for *Syndiniales* Dino‐Group I infections, these suggestions cannot be verified. Further insight is needed into *Syndiniales* Dino‐Group I—*Dinophyceae* and diatom interactions, as well as their potential negative relation with *Chloropicophyceae*.

The patterns observed for *Syndiniales* primarily seemed to be influenced by biotic interactions and do not appear homogeneous across the different water masses. All protist communities observed in this study did include the Dino‐Group‐I Clade‐I_ASV1 as the most dominant *Syndiniales* member, emphasising their ability to infect a wide host range (Anderson and Harvey 2020; Käse et al. 2021). Combined with the consequences that *Syndiniales* dominance could have for carbon cycling and the marine food web discussed earlier, this wide host range further supports the need to incorporate these parasitoids into ecosystem models. Finally, the observations from this study support the suggestion by Rizos et al. (2023) that *Syndiniales* could be potential candidates as marine bioindicators for environmental change, such as MHW events.

## 5 Conclusion

The extreme MHW of Austral summer 2017/2018 had profound impacts on the protist communities off the southeast coast of South New Zealand. We observed a significant reduction in protist alpha diversity in STW and the Front as well as a reduced beta diversity resulting in the absence of spatial structuring across the Southland Front. Changes in taxonomic composition of protist communities were caused by a transition from *Dinophyceae* to *Syndiniales* dominance in STW and the Front but were primarily driven by a surge in *Chloropicophyceae* in SASW. While temperature played a role in these shifts, it was not the sole driver, as other factors such as biotic interactions likely contributed to the changes observed. *Dinophyceae*, diatoms, and *Syndiniales* appeared to be connected through host‐parasitoid interactions, potentially influencing the community dynamics. Our study underscores the importance of considering *Syndiniales* in marine ecosystem models due to their significant impact on carbon cycling and food web dynamics. Overall, our study highlights the need to understand and monitor the effects of MHW events on marine protist communities and the role that *Syndiniales* play in this context.

## Author Contributions

**Hanneloor Heynderickx:** conceptualization, methodology, formal analysis, investigation, data curation, writing – original draft, visualization. **Ro Allen:** investigation, methodology, data curation, writing – review and editing. **Tina C. Summerfield:** resources, writing – review and editing, supervision. **Kim Currie:** investigation, data curation, writing – review and editing. **Linn J. Hoffmann:** conceptualization, writing – review and editing, supervision.

## Funding

This work was supported by the University of Otago PhD scholarship.

## Conflicts of Interest

The authors declare no conflicts of interest.

## Supporting information


**Table S1:** Macronutrient and chlorophyll‐*a* concentrations for each sampling station. The P5, P14 and P20 trips represent the summer cruises in January 2017, 2019, and 2020 respectively. The sampling trip (P10) done in January 2018 represents the marine heatwave. Water masses: Subtropical Water (STW), Southland Front (Front) and sub‐Antarctic Surface Water (SASW).
**Figure S1:** Continuous sea surface (2 m) temperature profiles for each sample cruise, as indicated on the right‐hand side of each plot. January 2018 represents the marine heatwave. Sample station temperature values measured at 10 m depth are added and coloured according to the characterised water mass. NW = Neritic Water, STW = Subtropical Water, Front = Southland Front, SASW = sub‐Antarctic Surface Water.
**Figure S2:** Continuous sea surface (2 m) salinity profiles for each sample cruise, as indicated on the right‐hand side of each plot. January 2018 represents the marine heatwave. Sample station salinity values measured at 10 m depth are added and coloured according to the characterised water mass. NW = Neritic Water, STW = Subtropical Water, Front = Southland Front, SASW = sub‐Antarctic Surface Water.
**Table S2:** Alpha diversity indices by water mass and heatwave status, with within water‐mass variations. ‘Non‐MHW’ represents the summer cruises in January 2017, 2019, and 2020. MHW = marine heatwave, represents the January 2018 cruise. Water masses: Subtropical Water (STW), Southland Front (Front) and sub‐Antarctic Surface Water (SASW). Bold cells (*) are significantly different from the corresponding non‐MHW value within that water mass, *p* < 0.05, Kruskal‐Wallis.
**Fig. S3:** Rarefaction curve with three candidate depths. Dashed lines = rarefaction depths of 14,883; 20,985 and 28,000 reads. Water masses: Subtropical Water (STW), Southland Front (Front) and sub‐Antarctic Surface Water (SASW).
**Table S3:** Rarefaction depth sensitivity of the primary PERMANOVA for the heatwave—water mass interaction term. Based on 1000 independent rarefaction draws per depth, following the repeated‐rarefying approach recommended by Cameron et al. (2021). *n* = samples with enough sequencing depth to be rarefied to the candidate depth. Mean R^2^ = average effect size across all 1000 draws at that depth. Min./Max *p*‐value = the highest and lowest *p*‐value across all 1.000 draws. % draws significant = fraction of 1000 independent draws that gives a statistically significant result.
**Table S4:** Rarefied and unrarefied normalisation comparison. The unrarefied dataset was normalised using a total‐sum‐scaled (TSS) transformation. R^2^‐ and *p*‐value refer to the heatwave—water mass interaction term (100,000 permutations). PERMDISP homogeneity of multivariate dispersion test was performed among water masses.
**Table S5:** Sensitivity of the primary PERMANOVA to dissimilarity metrics. All values are given at 100,000 permutations. PERMDISP *p*‐value is reported for water mass only.
**Table S6:** Jackknife robustness of the primary PERMANOVA. The jackknife analysis is a leave one sample out approach. The 10 most influential samples out of the full 28 are shown. All values are given at 100,000 permutations.
**Table S7:** dbRDA environmental variable selection stability. Jackknife (leave‐one‐sample‐out) forward selection repeated across 28 iterations (999 permutations per iteration); backward elimination performed once on the full dataset (100,000 permutations).
**Figure S4:** Class‐level taxonomical composition of protist communities in January 2017/2019/2020 (non‐MHW), and in January 2018 (MHW). STW: Subtropical Water, Front: Southland Front, and SASW: sub‐Antarctic Surface Water.
**Figure S5:** Heatmap of the top most abundant ASVs in Subtropical water. ‘No MHW’ represents the summer cruises in January 2017, 2019, and 2020. MHW = marine heatwave, represents the January 2018 cruise. All individual samples are shown along the x‐axis: *n* = 4 (No MHW) and *n* = 3 (MHW). The y‐axis represents the Division level and ASV number with the lowest possible assigned taxonomic rank down to genus. ASVs identified to ‘Dinoflagellata| Dino‐Group‐I‐Clade’ belong to the Syndiniales Class, while all other Dinoflagellata present here belong to the Dinophyceae Class. ASVs identified as ‘Chlorphyta| Chloroparvula’ belong to the Chloropicophyceae Class.
**Figure S6:** Heatmap of the top most abundant ASVs in the Southland Front. ‘No MHW’ represents the summer cruises in January 2017, 2019, and 2020. MHW = marine heatwave, represents the January 2018 cruise. All individual samples are shown along the x‐axis: *n* = 3 (No MHW) and *n* = 3 (MHW). The y‐axis represents the Division level and | ASV number with the lowest possible assigned taxonomic rank down to genus. ASVs identified to ‘Dinoflagellata| Dino‐Group‐I‐Clade’ belong to the Syndiniales Class, while all other Dinoflagellata present here belong to the Dinophyceae Class. ASVs identified as ‘Chlorphyta| Chloroparvula’ belong to the Chloropicophyceae Class.
**Figure S7:** Heatmap of the top most abundant ASVs in sub‐Antarctic surface water. ‘No MHW’ represents the summer cruises in January 2017, 2019, and 2020. MHW = marine heatwave, represents the January 2018 cruise. All individual samples are shown along the x‐axis: *n* = 13 (No MHW) and *n* = 3 (MHW). The y‐axis represents the Division level and | ASV number with the lowest possible assigned taxonomic rank down to genus. ASVs identified to ‘Dinoflagellata| Dino‐Group‐I‐Clade’ belong to the Syndiniales Class, while all other Dinoflagellata present here belong to the Dinophyceae Class. ASVs identified as ‘Chlorphyta| Chloroparvula’ and ‘Chlorphyta| Chloropicon’ belong to the Chloropicophyceae Class.

## Data Availability

The data that support the findings of this study are openly available in the European Nucleotide Archive (ENA) at EMBL‐EBI database under accession no. PRJEB76466 (www.ebi.ac.uk/ena/browser/view/PRJEB76466). Code for data analysis and figure generation is available in the GitHub repository (https://github.com/LoorH/MHW18S).

## References

[emi470430-bib-0001] Ahme, A. , I. V. Kirstein , C. L. Meunier , S. Wohlrab , and U. John . 2025. “Concurrent Global Change and Marine Heatwaves Disturb Phototrophic More Than Heterotrophic Protist Diversity.” Limnology and Oceanography Letters 10, no. 4: 473–484.

[emi470430-bib-0002] Allen, R. , T. C. Summerfield , K. Currie , P. W. Dillingham , and L. J. Hoffmann . 2020. “Distinct Processes Structure Bacterioplankton and Protist Communities Across an Oceanic Front.” Aquatic Microbial Ecology 85: 19–34. 10.3354/AME01938.

[emi470430-bib-0003] Allen, R. J. , T. C. Summerfield , B. P. Harvey , et al. 2021. “Species Turnover Underpins the Effect of Elevated CO_2_ on Biofilm Communities Through Early Succession.” Climate Change Ecology 2: 100017. 10.1016/J.ECOCHG.2021.100017.

[emi470430-bib-0004] Alves‐de‐Souza, C. , D. Pecqueur , E. Le Floc'h , et al. 2015. “Significance of Plankton Community Structure and Nutrient Availability for the Control of Dinoflagellate Blooms by Parasites: A Modeling Approach.” PLoS One 10, no. 6: e0127623. 10.1371/JOURNAL.PONE.0127623. PMC4452582 26030411

[emi470430-bib-0005] Anderson, M. J. 2006. “Distance‐Based Tests for Homogeneity of Multivariate Dispersions.” Biometrics 62, no. 1: 245–253. 10.1111/j.1541-0420.2005.00440.x. 16542252

[emi470430-bib-0006] Anderson, M. J. 2017. “Permutational Multivariate Analysis of Variance (PERMANOVA).” In Wiley StatsRef: Statistics Reference Online, edited by N. Balakrishnan , T. Colton , B. Everitt , W. Piegorsch , F. Ruggeri , and J. L. Teugels . 10.1002/9781118445112.stat07841.

[emi470430-bib-0007] Anderson, S. R. , and E. L. Harvey . 2020. “Temporal Variability and Ecological Interactions of Parasitic Marine Syndiniales in Coastal Protist Communities.” mSphere 5: e00209‐20. 10.1128/mSphere.00209-20. PMC7253595 32461270

[emi470430-bib-0008] Bachvaroff, T. R. , S. Kim , L. Guillou , C. F. Delwiche , and D. W. Coats . 2012. “Molecular Diversity of the Syndinean Genus *Euduboscquella* Based on Single‐Cell PCR Analysis.” Applied and Environmental Microbiology 78: 334–345. 10.1128/AEM.06678-11. PMC3255722 22081578

[emi470430-bib-0009] Banse, K. 1996. “Low Seasonality of Low Concentrations of Surface Chlorophyll in the Subantarctic Water Ring: Underwater Irradiance, Iron, or Grazing?” Progress in Oceanography 37: 241–291. 10.1016/S0079-6611(96)00006-7.

[emi470430-bib-0010] Banzon, V. , T. M. Smith , M. Steele , B. Huang , and H. M. Zhang . 2020. “Improved Estimation of Proxy Sea Surface Temperature in the Arctic.” Journal of Atmospheric and Oceanic Technology 37: 341–349. 10.1175/JTECH-D-19-0177.1.

[emi470430-bib-0011] Behrenfeld, M. J. , R. T. O'Malley , D. A. Siegel , et al. 2006. “Climate‐Driven Trends in Contemporary Ocean Productivity.” Nature 444, no. 7120: 752–755. 10.1038/nature05317. 17151666

[emi470430-bib-0012] Berdjeb, L. , A. Parada , D. M. Needham , and J. A. Fuhrman . 2018. “Short‐Term Dynamics and Interactions of Marine Protist Communities During the Spring–Summer Transition.” ISME Journal 12, no. 8: 1907–1917. 10.1038/s41396-018-0097-x. PMC6052004 29599520

[emi470430-bib-0013] Bettim, A. L. , M. Z. Marochi , R. Metri , and P. D. Guilherme . 2025. “Impacts of Marine Heatwaves on Benthic Estuarine Populations.” Climatic Change 178, no. 10: 181.

[emi470430-bib-0014] Bjorbækmo, M. F. M. , A. Evenstad , L. L. Røsæg , A. K. Krabberød , and R. Logares . 2020. “The Planktonic Protist Interactome: Where Do We Stand After a Century of Research?” ISME Journal 14, no. 2: 544–559. 10.1038/s41396-019-0542-5. PMC6976576 31685936

[emi470430-bib-0015] Brodeur, R. D. , T. D. Auth , and A. J. Phillips . 2019. “Major Shifts in Pelagic Micronekton and Macrozooplankton Community Structure in an Upwelling Ecosystem Related to an Unprecedented Marine Heatwave.” Frontiers in Marine Science 6: 212. 10.3389/FMARS.2019.00212/BIBTEX.

[emi470430-bib-0016] Cai, R. , E. Kayal , C. Alves‐de‐Souza , et al. 2020. “Cryptic Species in the Parasitic Amoebophrya Species Complex Revealed by a Polyphasic Approach.” Scientific Reports 10, no. 1: 1–11. 10.1038/s41598-020-59524-z. PMC7018713 32054950

[emi470430-bib-0017] Callahan, B. J. , P. J. McMurdie , M. J. Rosen , et al. 2016a. “DADA2: High‐Resolution Sample Inference From Illumina Amplicon Data.” Nature Methods 13, no. 7: 581–583. 10.1038/nmeth.3869. PMC4927377 27214047

[emi470430-bib-0018] Callahan, B. J. , K. Sankaran , J. A. Fukuyama , P. J. McMurdie , and S. P. Holmes . 2016b. “Bioconductor Workflow for Microbiome Data Analysis: From Raw Reads to Community Analyses.” F1000Research 5: 1492. 10.12688/f1000research.8986.2. PMC4955027 27508062

[emi470430-bib-0019] Cameron, E. S. , P. J. Schmidt , B. J. M. Tremblay , B. J. M. Tremblay , M. B. Emelko , and K. M. Müller . 2021. “Enhancing Diversity Analysis by Repeatedly Rarefying Next Generation Sequencing Data Describing Microbial Communities.” Scientific Reports 11: 22302. 10.1038/s41598-021-01636-1. PMC8595385 34785722

[emi470430-bib-0020] Castro, N. , P. Ramalhosa , E. Cacabelos , J. L. Costa , J. Canning‐Clode , and I. Gestoso . 2021. “Winners and Losers: Prevalence of Non‐Indigenous Species Under Simulated Marine Heatwaves and High Propagule Pressure.” Marine Ecology Progress Series 668: 21–38. 10.3354/MEPS13710.

[emi470430-bib-0021] Cavole, L. M. , A. Demko , R. E. Diner , et al. 2016. “Biological Impacts of the 2013–2015 Warm‐Water Anomaly in the Northeast Pacific: Winners, Losers, and the Future.” Oceanography 29: 273–285. 10.5670/OCEANOG.2016.32.

[emi470430-bib-0022] Chambouvet, A. , C. Alves‐de‐Souza , V. Cueff , D. Marie , S. Karpov , and L. Guillou . 2011. “Interplay Between the Parasite *Amoebophrya* sp. (Alveolata) and the Cyst Formation of the Red Tide Dinoflagellate *Scrippsiella trochoidea* .” Protist 162: 637–649. 10.1016/J.PROTIS.2010.12.001. 21349764

[emi470430-bib-0023] Chambouvet, A. , P. Morin , D. Marie , and L. Guillou . 2008. “Control of Toxic Marine Dinoflagellate Blooms by Serial Parasitic Killers.” Science 322: 1254–1257. 10.1126/SCIENCE.1164387. 19023082

[emi470430-bib-0024] Chauhan, A. , P. A. H. Smith , F. Rodrigues , A. Christensen , M. St. John , and P. Mariani . 2023. “Distribution and Impacts of Long‐Lasting Marine Heat Waves on Phytoplankton Biomass.” Frontiers in Marine Science 10: 1177571. 10.3389/fmars.2023.1177571.

[emi470430-bib-0025] Clarke, K. R. 1993. “Non‐Parametric Multivariate Analyses of Changes in Community Structure.” Australian Journal of Ecology 18: 117–143. 10.1111/J.1442-9993.1993.TB00438.X.

[emi470430-bib-0026] Clarke, L. J. , S. Bestley , A. Bissett , and B. E. Deagle . 2019. “A Globally Distributed Syndiniales Parasite Dominates the Southern Ocean Micro‐Eukaryote Community Near the Sea‐Ice Edge.” ISME Journal 13, no. 3: 734–737. 10.1038/s41396-018-0306-7. PMC6461979 30367123

[emi470430-bib-0027] Coats, D. W. , and M. G. Park . 2002. “Parasitism of Photosynthetic Dinoflagellates by Three Strains of *Amoebophrya* (Dinophyta): Parasite Survival, Infectivity, Generation Time, and Host Specificity.” Journal of Phycology 38: 520–528. 10.1046/J.1529-8817.2002.01200.X.

[emi470430-bib-0028] Countway, P. D. , R. J. Gast , P. Savai , and D. A. Caron . 2005. “Protistan Diversity Estimates Based on 18S rDNA From Seawater Incubations in the Western North Atlantic.” Journal of Eukaryotic Microbiology 52: 95–106. 10.1111/J.1550-7408.2005.05202006.X. 15817114

[emi470430-bib-0029] Crane, K. W. , and J. P. Grover . 2010. “Coexistence of Mixotrophs, Autotrophs, and Heterotrophs in Planktonic Microbial Communities.” Journal of Theoretical Biology 262: 517–527. 10.1016/J.JTBI.2009.10.027. 19878684

[emi470430-bib-0030] Currie, K. I. , M. R. Reid , and K. A. Hunter . 2011. “Interannual Variability of Carbon Dioxide Drawdown by Subantarctic Surface Water Near New Zealand.” Biogeochemistry 104: 23–34. 10.1007/s10533-009-9355-3.

[emi470430-bib-0031] De Vargas, C. , S. Audic , N. Henry , et al. 2015. “Ocean Plankton. Eukaryotic Plankton Diversity in the Sunlit Ocean.” Science 348, no. 6237: 1261605. 10.1126/SCIENCE.1261605. 25999516

[emi470430-bib-0032] Eglaine, Z. , J. Courboulès , F. Cipolletta , et al. 2025. “Effects of a Simulated Marine Heatwave on the Structure and Composition of Mediterranean Plankton in a Mesocosm Study.” PLoS One 20, no. 11: e0337112. 10.1371/journal.pone.0337112 PMC12637984 41270089

[emi470430-bib-0033] Falkowski, P. G. , T. Fenchel , and E. F. Delong . 2008. “The Microbial Engines That Drive Earth's Biogeochemical Cycles.” Science (1979) 320: 1034–1039. 10.1126/science.1153213. 18497287

[emi470430-bib-0034] Farhat, S. , P. Le , E. Kayal , et al. 2021. “Rapid Protein Evolution, Organellar Reductions, and Invasive Intronic Elements in the Marine Aerobic Parasite Dinoflagellate *Amoebophrya* spp.” BMC Biology 19: 1–21. 10.1186/S12915-020-00927-9. PMC7789003 33407428

[emi470430-bib-0035] Garrabou, J. , R. Coma , N. Bensoussan , et al. 2009. “Mass Mortality in Northwestern Mediterranean Rocky Benthic Communities: Effects of the 2003 Heat Wave.” Global Change Biology 15: 1090–1103. 10.1111/J.1365-2486.2008.01823.X.

[emi470430-bib-0036] Gong, W. , and A. Marchetti . 2019. “Estimation of 18 S Gene Copy Number in Marine Eukaryotic Plankton Using a Next‐Generation Sequencing Approach.” Frontiers in Marine Science 6: 219.

[emi470430-bib-0037] Griffith, J. C. , W. G. Lee , D. A. Orlovich , and T. C. Summerfield . 2017. “Contrasting Bacterial Communities in Two Indigenous *Chionochloa* (Poaceae) Grassland Soils in New Zealand.” PLoS One 12: e0179652. 10.1371/JOURNAL.PONE.0179652. PMC5489180 28658306

[emi470430-bib-0038] Guillou, L. , D. Bachar , S. Audic , et al. 2012. “The Protist Ribosomal Reference Database (PR2): A Catalog of Unicellular Eukaryote Small Sub‐Unit rRNA Sequences With Curated Taxonomy.” Nucleic Acids Research 41, no. D1: D597–D604. 10.1093/NAR/GKS1160. PMC3531120 23193267

[emi470430-bib-0039] Guillou, L. , M. Viprey , A. Chambouvet , et al. 2008. “Widespread Occurrence and Genetic Diversity of Marine Parasitoids Belonging to *Syndiniales* (Alveolata).” Environmental Microbiology 10: 3349–3365. 10.1111/J.1462-2920.2008.01731.X. 18771501

[emi470430-bib-0040] Hatcher, M. J. , and A. M. Dunn . 2011. Parasites in Ecological Communities: From Interactions to Ecosystems. Cambridge University Press. 10.1017/CBO9780511987359.

[emi470430-bib-0041] Hawke, D. J. 1989. “Hydrology and Near‐Surface Nutrient Distribution Along the South Otago Continental Shelf, New Zealand, in Summer and Winter 1986.” New Zealand Journal of Marine and Freshwater Research 23: 411–420. 10.1080/00288330.1989.9516377.

[emi470430-bib-0042] Heath, R. A. 1981. “Oceanic Fronts Around Southern New Zealand.” Deep Sea Research Part A, Oceanographic Research Papers 28: 547–560. 10.1016/0198-0149(81)90116-3.

[emi470430-bib-0043] Heynderickx, H. C. D. 2022. Marine Microbial Community Diversity and Responses to Environmental Change (Doctoral Dissertation, University of Otago).

[emi470430-bib-0044] Hobday, A. J. , L. V. Alexander , S. E. Perkins , et al. 2016. “A Hierarchical Approach to Defining Marine Heatwaves.” Progress in Oceanography 141: 227–238. 10.1016/J.POCEAN.2015.12.014.

[emi470430-bib-0045] Hobday, A. J. , E. C. Oliver , A. S. Gupta , et al. 2018. “Categorizing and Naming Marine Heatwaves.” Oceanography 31: 162–173. 10.5670/OCEANOG.2018.205.

[emi470430-bib-0046] Hopkins, J. , A. G. P. Shaw , and P. Challenor . 2010. “The Southland Front, New Zealand: Variability and ENSO Correlations.” Continental Shelf Research 30: 1535–1548. 10.1016/j.csr.2010.05.016.

[emi470430-bib-0047] Hughes, T. P. , J. T. Kerry , A. H. Baird , et al. 2018. “Global Warming Transforms Coral Reef Assemblages.” Nature 556, no. 7702: 492–496. 10.1038/s41586-018-0041-2. 29670282

[emi470430-bib-0048] IPCC, F. C . 2012. IPCC: Managing the Risks of Extreme Events and Disasters to Advance Climate Change Adaptation. A Special Report of Working Groups I and II of the Intergovernmental Panel on Climate Change 582. 10.1136/jech-2012-201045 22766781

[emi470430-bib-0049] Jillett, J. B. 1969. “Seasonal Hydrology of Waters Off the Otago Peninsula, South‐Eastern New Zealand.” New Zealand Journal of Marine and Freshwater Research 3: 349–375. 10.1080/00288330.1969.9515303.

[emi470430-bib-0050] Jones, K. N. , K. I. Currie , C. M. McGraw , and K. A. Hunter . 2013. “The Effect of Coastal Processes on Phytoplankton Biomass and Primary Production Within the Near‐Shore Subtropical Frontal Zone.” Estuarine, Coastal and Shelf Science 124: 44–55. 10.1016/J.ECSS.2013.03.003.

[emi470430-bib-0051] Käse, L. , K. Metfies , S. Neuhaus , M. Boersma , K. H. Wiltshire , and A. C. Kraberg . 2021. “Host‐Parasitoid Associations in Marine Planktonic Time Series: Can Metabarcoding Help Reveal Them?” PLoS One 16: e0244817. 10.1371/JOURNAL.PONE.0244817. PMC7790432 33411833

[emi470430-bib-0052] Laget, M. , L. Drago , T. Panaïotis , et al. 2024. “Global Census of the Significance of Giant Mesopelagic Protists to the Marine Carbon and Silicon Cycles.” Nature Communications 15, no. 1: 3341. 10.1038/s41467-024-47651-4 PMC11058905 38684684

[emi470430-bib-0053] Legendre, P. , and M. J. Anderson . 1999. “Distance‐Based Redundancy Analysis: Testing Multispecies Responses in Multifactorial Ecological Experiments.” Ecological Monographs 69, no. 1: 1–24.

[emi470430-bib-0054] Lima‐Mendez, G. , K. Faust , N. Henry , et al. 2015. “Determinants of Community Structure in the Global Plankton Interactome.” Science 348, no. 6237: 1262073. 10.1126/science.1262073 25999517

[emi470430-bib-0055] Liu, Z. , L. Yang , L. Yan , et al. 2022. “Alteration of Alpha and Beta Diversity in Nekton Community by Extreme Marine Heatwave Events: An Example From the East China Sea.” Frontiers in Marine Science 9: 1036047. 10.3389/FMARS.2022.1036047/BIBTEX.

[emi470430-bib-0056] Loeblich, A. R. 1976. “Dinoflagellate Evolution: Speculation and Evidence.” Journal of Protozoology 23, no. 1: 13–28. 10.1111/j.1550-7408.1976.tb05241.x 944775

[emi470430-bib-0057] Massana, R. , A. Gobet , S. Audic , et al. 2015. “Marine Protist Diversity in European Coastal Waters and Sediments as Revealed by High‐Throughput Sequencing.” Environmental Microbiology 17: 4035–4049. 10.1111/1462-2920.12955. 26119494

[emi470430-bib-0058] McMurdie, P. J. , and S. Holmes . 2013. “Phyloseq: An R Package for Reproducible Interactive Analysis and Graphics of Microbiome Census Data.” PLoS One 8: e61217. 10.1371/JOURNAL.PONE.0061217. PMC3632530 23630581

[emi470430-bib-0059] Moore, J. K. , S. C. Doney , D. M. Glover , and I. Y. Fung . 2001. “Iron Cycling and Nutrient‐Limitation Patterns in Surface Waters of the World Ocean.” Deep Sea Research Part II: Topical Studies in Oceanography 49: 463–507. 10.1016/S0967-0645(01)00109-6.

[emi470430-bib-0060] Moran, M. A. , F. X. Ferrer‐González , H. Fu , et al. 2022. “The Ocean's Labile DOC Supply Chain.” Limnology and Oceanography 67: 1007–1021. 10.1002/LNO.12053.

[emi470430-bib-0061] Na, S. I. , H. J. Seo , and S. H. Jang . 2026. “Integrative Insights Into Marine Protist Assemblages Between Surface and Deep Chlorophyll Maximum.” Frontiers in Marine Science 13: 1756144.

[emi470430-bib-0062] Nagarkar, M. , and B. Palenik . 2023. “Diversity and Putative Interactions of Parasitic Alveolates Belonging to Syndiniales at a Coastal Pacific Site.” Environmental Microbiology Reports 15: 157–169. 10.1111/1758-2229.13138. PMC10464665 36779254

[emi470430-bib-0063] NIWA . 2019. NIWA National Climate Summary 2018: New Zealand's Equal‐2nd Warmest Year on Record.

[emi470430-bib-0064] Oksanen, J. 2020. Package “vegan” Title Community Ecology Package Version 2.5–7.

[emi470430-bib-0065] Oliver, E. C. , M. T. Burrows , M. G. Donat , et al. 2019. “Projected Marine Heatwaves in the 21st Century and the Potential for Ecological Impact.” Frontiers in Marine Science 6: 734.

[emi470430-bib-0066] R Core Team . 2021. R: A Language and Environment for Statistical Computing. R Foundation for Statistical Computing. https://www.R‐project.org/.

[emi470430-bib-0067] Rasconi, S. , K. Winter , and M. J. Kainz . 2017. “Temperature Increase and Fluctuation Induce Phytoplankton Biodiversity Loss – Evidence From a Multi‐Seasonal Mesocosm Experiment.” Ecology and Evolution 7: 2936–2946. 10.1002/ECE3.2889. PMC5415537 28479993

[emi470430-bib-0068] Remy, M. , H. Hillebrand , and S. Flöder . 2017. “Stability of Marine Phytoplankton Communities Facing Stress Related to Global Change: Interactive Effects of Heat Waves and Turbidity.” Journal of Experimental Marine Biology and Ecology 497: 219–229. 10.1016/J.JEMBE.2017.10.002.

[emi470430-bib-0069] Reynolds, R. W. , T. M. Smith , C. Liu , D. B. Chelton , K. S. Casey , and M. G. Schlax . 2007. “Daily High‐Resolution‐Blended Analyses for Sea Surface Temperature.” Journal of Climate 20: 5473–5496. 10.1175/2007JCLI1824.1.

[emi470430-bib-0070] Rizos, I. , P. Debeljak , T. Finet , et al. 2023. “Beyond the Limits of the Unassigned Protist Microbiome: Inferring Large‐Scale Spatio‐Temporal Patterns of Syndiniales Marine Parasites.” ISME Communications 3, no. 1: 1–11. 10.1038/s43705-022-00203-7. PMC9975217 36854980

[emi470430-bib-0071] Sarmiento, J. L. , N. Gruber , M. A. Brzezinski , and J. P. Dunne . 2004. “High‐Latitude Controls of Thermocline Nutrients and Low Latitude Biological Productivity.” Nature 427, no. 6969: 56–60. 10.1038/nature02127. 14702082

[emi470430-bib-0072] Sassenhagen, I. , S. Irion , L. Jardillier , D. Moreira , and U. Christaki . 2020. “Protist Interactions and Community Structure During Early Autumn in the Kerguelen Region (Southern Ocean).” Protist 171: 125709. 10.1016/J.PROTIS.2019.125709. 32004979

[emi470430-bib-0073] Scheibling, R. E. , and P. Gagnon . 2009. “Temperature‐Mediated Outbreak Dynamics of the Invasive Bryozoan *Membranipora membranacea* in Nova Scotian Kelp Beds.” Marine Ecology Progress Series 390: 1–13. 10.3354/MEPS08207.

[emi470430-bib-0074] Schlegel, R. W. 2020. Marine Heatwave Tracker. 10.5281/zenodo.3787872.

[emi470430-bib-0075] Sen Gupta, A. , M. Thomsen , J. A. Benthuysen , et al. 2020. “Drivers and Impacts of the Most Extreme Marine Heatwave Events.” Scientific Reports 10, no. 1: 1–15. 10.1038/s41598-020-75445-3. PMC7653907 33168858

[emi470430-bib-0076] Shadrin, A. M. , M. V. Kholodova , and D. S. Pavlov . 2010. “Geographic Distribution and Molecular Genetic Identification of the Parasite of the Genus *Ichthyodinium* Causing Mass Mortality of Fish Eggs and Larvae in Coastal Waters of Vietnam.” Doklady Biological Sciences 432: 220–223. 10.1134/S0012496610030154/METRICS. 20665159

[emi470430-bib-0078] Smale, D. A. , J. D. Taylor , S. H. Coombs , G. Moore , and M. Cunliffe . 2017. “Community Responses to Seawater Warming Are Conserved Across Diverse Biological Groupings and Taxonomic Resolutions.” Proceedings of the Royal Society B: Biological Sciences 284: 20170534. 10.1098/rspb.2017.0534. PMC5597821 28878056

[emi470430-bib-0077] Smale, D. A. , T. Wernberg , E. C. Oliver , et al. 2019. “Marine Heatwaves Threaten Global Biodiversity and the Provision of Ecosystem Services.” Nature Climate Change 9: 306–312. 10.1038/s41558-019-0412-1.

[emi470430-bib-0079] Smith, J. G. , C. M. Free , C. Lopazanski , et al. 2023. “A Marine Protected Area Network Does Not Confer Community Structure Resilience to a Marine Heatwave Across Coastal Ecosystems.” Global Change Biology 29: 5634–5651. 10.1111/gcb.16862. 37439293

[emi470430-bib-0080] Smith, K. E. , M. T. Burrows , A. J. Hobday , et al. 2023. “Biological Impacts of Marine Heatwaves.” Annual Review of Marine Science 15, no. 1: 119–145. 10.1146/annurev-marine-032122-121437 35977411

[emi470430-bib-0081] Soulié, T. , F. Vidussi , S. Mas , and B. Mostajir . 2022. “Functional Stability of a Coastal Mediterranean Plankton Community During an Experimental Marine Heatwave.” Frontiers in Marine Science 9: 68. 10.3389/FMARS.2022.831496/BIBTEX.

[emi470430-bib-0082] Stoeck, T. , D. Bass , M. Nebel , et al. 2010. “Multiple Marker Parallel Tag Environmental DNA Sequencing Reveals a Highly Complex Eukaryotic Community in Marine Anoxic Water.” Molecular Ecology 19: 21–31. 10.1111/j.1365-294X.2009.04480.x. 20331767

[emi470430-bib-0083] Thompson, A. R. , N. J. Ben‐Aderet , N. M. Bowlin , D. Kacev , R. Swalethorp , and W. Watson . 2022. “Putting the Pacific Marine Heatwave Into Perspective: The Response of Larval Fish Off Southern California to Unprecedented Warming in 2014–2016 Relative to the Previous 65 Years.” Global Change Biology 28: 1766–1785. 10.1111/GCB.16010. 34951510

[emi470430-bib-0084] Thomsen, M. S. , L. Mondardini , T. Alestra , et al. 2019. “Local Extinction of Bull Kelp (*Durvillaea* spp.) due to a Marine Heatwave.” Frontiers in Marine Science 6: 1–10. 10.3389/FMARS.2019.00084/FULL.

[emi470430-bib-0085] Thomson, J. A. , D. A. Burkholder , M. R. Heithaus , et al. 2015. “Extreme Temperatures, Foundation Species, and Abrupt Ecosystem Change: An Example From an Iconic Seagrass Ecosystem.” Global Change Biology 21: 1463–1474. 10.1111/GCB.12694. 25145694

[emi470430-bib-0086] Warton, D. I. , S. T. Wright , and Y. Wang . 2012. “Distance‐Based Multivariate Analyses Confound Location and Dispersion Effects.” Methods in Ecology and Evolution 3: 89–101. 10.1111/J.2041-210X.2011.00127.X.

[emi470430-bib-0087] Wernberg, T. , M. S. Thomsen , M. T. Burrows , et al. 2025. “Marine Heatwaves as Hot Spots of Climate Change and Impacts on Biodiversity and Ecosystem Services.” Nature Reviews Biodiversity 1, no. 7: 461–479.

[emi470430-bib-0088] Wickham, H. 2016. ggplot2: Elegant Graphics for Data Analysis. Springer‐Verlag.

[emi470430-bib-0089] Worden, A. Z. , M. J. Follows , S. J. Giovannoni , S. Wilken , A. E. Zimmerman , and P. J. Keeling . 2015. “Rethinking the Marine Carbon Cycle: Factoring in the Multifarious Lifestyles of Microbes.” Science 347, no. 6223: 1257594. 10.1126/science.1257594. 25678667

[emi470430-bib-0090] Zhan, W. , M. Feng , Y. Zhang , X. Shen , H. Zhan , and Q. He . 2024. “Reduced and Smaller Phytoplankton During Marine Heatwaves in Eastern Boundary Upwelling Systems.” Communications Earth & Environment 5: 629. 10.1038/s43247-024-01805-w.

[emi470430-bib-0091] Zhang, Y. , G. Gao , H. Xue , and K. Gao . 2025. “Reduced Primary Productivity and Notable Resilience of Phytoplankton Community in the Coastal Water of Southern China Under a Marine Heatwave.” Environmental Research 264: 120286. 10.1016/j.envres.2024.120286. 39491607

