# Supplementary material for: Shift Towards Syndiniales Prevalence in Protist Communities During a Marine Heatwave
Source: Environ Microbiol Rep. 2026 Oct 11;18(5):e70430. doi: 10.1111/1758-2229.70430 (PMC13655307; doi:10.1111/1758-2229.70430)
Supplement: Supplementary file 1 [file EMI4-18-e70430-s001.docx]

**Supporting Information for:** **Shift towards Syndiniales prevalence in protist communities during a marine heatwave**

Hanneloor Heynderickx^1,3,§,*^ 0000-0002-5611-837X, Ro Allen^1^ 0000-0002-7109-3721, Tina C. Summerfield^1^ 0000-0003-1878-7019, Kim Currie^2^ 0000-0003-4252-194X, Linn J. Hoffmann^1^ 0000-0003-0242-4686

^1^Department of Botany, University of Otago, Dunedin 9016, New Zealand

^2^National Institute of Water and Atmospheric Research, Dunedin, New Zealand
^3,§^Marine Observation Centre, Flanders Marine institute, Oostende 8400, Belgium

^*^Corresponding author: linn.hoffmann@otago.ac.nz, Department of Botany, University of Otago, Dunedin, New Zealand^[1]^

**Section A. Environmental variables**

**Table S1:** Macronutrient and chlorophyll-*a* concentrations for each sampling station. The P5, P14 and P20 trips represent the summer cruises in January 2017, 2019, and 2020 respectively. The sampling trip (P10) done in January 2018 represents the marine heatwave. Water masses: Subtropical Water (STW), Southland Front (Front) and sub-Antarctic Surface Water (SASW).

| Trip | SampleID | Date | Water mass | Phosphate (µM) | Nitrate (µM) | Silicate (µM) | Chla (mg m⁻³) |
| --- | --- | --- | --- | --- | --- | --- | --- |
| P5 | P5S1 | 17-Jan | STW | 0.30 | 2.90 | 1.37 | 2.06 |
| P5 | P5S3 | 17-Jan | Front | 0.46 | 5.56 | 1.57 | 1.25 |
| P5 | P5S5 | 17-Jan | SASW | 0.66 | 9.50 | 0.38 | 0.85 |
| P5 | P5S6 | 17-Jan | SASW | 0.61 | 9.07 | 0.37 | 0.28 |
| P5 | P5S7 | 17-Jan | SASW | 0.67 | 10.0 | 0.40 | 0.28 |
| P5 | P5S8 | 17-Jan | SASW | 0.67 | 10.5 | 0.49 | 0.21 |
| P10 | P10S1 | 18-Jan | STW | 0.17 | 1.37 | 1.14 | 0.25 |
| P10 | P10S2 | 18-Jan | STW | 0.21 | 1.79 | 1.03 | 0.29 |
| P10 | P10S3 | 18-Jan | STW | 0.10 | 0.47 | 0.71 | 0.28 |
| P10 | P10S4 | 18-Jan | Front | 0.41 | 5.57 | 0.51 | 0.21 |
| P10 | P10S5 | 18-Jan | Front | 0.42 | 6.00 | 0.61 | 0.21 |
| P10 | P10S6 | 18-Jan | Front | 0.41 | 5.57 | 0.61 | 0.22 |
| P10 | P10S7 | 18-Jan | SASW | 0.44 | 6.57 | 0.43 | 0.23 |
| P10 | P10S8 | 18-Jan | SASW | 0.43 | 6.45 | 0.45 | 0.17 |
| P14 | P14S2 | 19-Jan | STW | 0.19 | 0.47 | 0.72 | 1.19 |
| P14 | P14S3 | 19-Jan | STW | 0.31 | 1.33 | 0.40 | 1.36 |
| P14 | P14S4 | 19-Jan | Front | 0.28 | 1.65 | 0.44 | 1.69 |
| P14 | P14S5 | 19-Jan | Front | 0.67 | 7.64 | 1.06 | 0.51 |
| P14 | P14S6 | 19-Jan | SASW | 0.79 | 8.71 | 1.06 | 0.27 |
| P14 | P14S7 | 19-Jan | SASW | 0.91 | 9.43 | 1.14 | 0.22 |
| P14 | P14S8 | 19-Jan | SASW | 0.78 | 8.82 | 0.71 | 0.28 |
| P20 | P20S2 | 20-Jan | STW | 0.30 | 1.30 | 1.40 | 0.90 |
| P20 | P20S3 | 20-Jan | SASW | 0.57 | 8.21 | 0.37 | 0.17 |
| P20 | P20S4 | 20-Jan | SASW | 0.57 | 8.36 | 0.32 | 0.19 |
| P20 | P20S5 | 20-Jan | SASW | 0.55 | 8.00 | 0.24 | 0.14 |
| P20 | P20S6 | 20-Jan | SASW | 0.51 | 7.29 | 0.07 | 0.14 |
| P20 | P20S7 | 20-Jan | SASW | 0.49 | 7.14 | 0.12 | 0.27 |
| P20 | P20S8 | 20-Jan | SASW | 0.51 | 7.40 | 0.06 | 0.27 |

**Fig. S1:** Continuous sea surface (2m) temperature profiles for each sample cruise, as indicated on the right-hand side of each plot. January 2018 represents the marine heatwave. Sample station temperature values measured at 10 m depth are added and coloured according to the characterised water mass. NW = Neritic Water, STW = Subtropical Water, Front = Southland Front, SASW = sub-Antarctic Surface Water.

**Fig. S2:** Continuous sea surface (2 m) salinity profiles for each sample cruise, as indicated on the right-hand side of each plot. January 2018 represents the marine heatwave. Sample station salinity values measured at 10 m depth are added and coloured according to the characterised water mass. NW = Neritic Water, STW = Subtropical Water, Front = Southland Front, SASW = sub-Antarctic Surface Water.

**Section B. Alpha diversity**

**Table S2.** Alpha diversity indices by water mass and heatwave status, with within water-mass variations. ‘Non-MHW’ represents the summer cruises in January 2017, 2019, and 2020. MHW = marine heatwave, represents the January 2018 cruise. Water masses: Subtropical Water (STW), Southland Front (Front) and sub-Antarctic Surface Water (SASW). Bold cells (*) are significantly different from the corresponding non-MHW value within that water mass, *p* < 0.05, Kruskal-Wallis.

| **Water mass** | **Status** | **Shannon  (mean ± SD)** | **Hill q=0 richness (mean ± SD)** | **Pielou  (mean ± SD)** | **n** |
| --- | --- | --- | --- | --- | --- |
| STW | Non-MHW | 3.91 ± 0.31 | 217.8 ± 57.2 | 0.73 ± 0.05 | 4 |
| STW | MHW | **1.78 ± 0.42*** | 150.7 ± 53.6 (n.s.) | **0.36 ± 0.06*** | 3 |
| Front | Non-MHW | 5.16 ± 0.12 | 499.0 ± 11.8 | 0.83 ± 0.02 | 3 |
| Front | MHW | **3.26 ± 0.19*** | **280.0 ± 47.5*** | **0.58 ± 0.02*** | 3 |
| SASW | Non-MHW | 4.03 ± 0.96 | 316.8 ± 134.7 | 0.70 ± 0.12 | 13 |
| SASW | MHW | 2.74 ± 0.26 (n.s.) | 188.0 ± 5.7 (n.s.) | 0.52 ± 0.05 (n.s.) | 2 |

**Section C. Robustness and sensitivity analyses**

1. **Rarefaction depth and normalisation sensitivity**

To confirm that the selected rarefaction depth (14,883 reads) and the decision to rarefy did not bias the primary PERMANOVA result (significant heatwave – water mass interaction), the analysis was repeated at three candidate depths (14,883; 20,985 and 28,000 reads; Fig S3), spanning the range of sequencing depths observed in the full dataset. Each depth had 1,000 independent rarefaction draws. This validation step followed the repeated-rarefying approach recommended by Cameron et al. (2021) to characterise the variability introduced by library-size normalization (Table S3). At 14,883 and 20,985 reads all 28 samples retained sufficient sequencing depth to be included. The heatwave - water mass interaction term remained statistically significant (*p* < 0.05) across these two depths in 100% of the 1,000 independent rarefaction draws, with negligible variation in effect size across draws (SD ≤ 0.0005). This indicates that the result does not depend on the specific random subsample of reads retained during rarefaction at either depth. At 28,000 reads, the proportion of significant draws fell sharply to 1.7%. This depth necessarily excluded five samples whose total sequencing depth fell below this threshold, reducing the dataset from 28 to 23 samples. This reduction is not random with respect to the result being tested: a separate jackknife analysis (Table S6) identified two of these five excluded samples as the most statistically influential individual samples for the heatwave - water mass interaction term in the full dataset. Their combined exclusion at this depth, alongside three additional samples, therefore, removes a disproportionate part of the evidence supporting the interaction, reducing statistical power even though the underlying effect size remained essentially unchanged (mean R² = 0.098, compared to 0.102 at the two lower depths). This pattern is consistent with a sample-size effect arising from the depth-dependent exclusion of specific, highly informative samples, rather than an underlying sensitivity of the result to rarefaction depth itself.

As a further check on the decision to rarefy at all, the primary PERMANOVA analysis was additionally repeated using total-sum-scaling (proportion-based normalisation without depth equalisation). Results remained steady under both Bray-Curtis and Hellinger-Euclidean dissimilarities (Table S4) with significant *p*-values throughout for the interaction term and non-significant values (p > 0.05) for PERMDISP (dispersions) under either normalisation or metric.

**Fig. S3:** Rarefaction curve with three candidate depths. Dashed lines = rarefaction depths of 14,883; 20,985 and 28,000 reads. Water masses: Subtropical Water (STW), Southland Front (Front) and sub-Antarctic Surface Water (SASW).

**Table S3.** Rarefaction depth sensitivity of the primary PERMANOVA for the heatwave - water mass interaction term. Based on 1,000 independent rarefaction draws per depth, following the repeated-rarefying approach recommended by Cameron et al. (2021). n = samples with enough sequencing depth to be rarefied to the candidate depth. Mean R² = average effect size across all 1,000 draws at that depth. Min./Max *p*-value = the highest and lowest *p*-value across all 1.000 draws. % draws significant = fraction of 1,000 independent draws that gives a statistically significant result.

| **Depth (reads)** | **n samples** | **Mean  R²-value** | **SD (R²)** | **Min.  *p*-value** | **Max. *p*-value** | **% draws significant  (*p* < 0.05)** |
| --- | --- | --- | --- | --- | --- | --- |
| 14,883 | 28 | 0.102 | 0.00047 | 0.001 | 0.014 | 100% |
| 20,985 | 28 | 0.102 | 0.00032 | 0.001 | 0.015 | 100% |
| 28,000 | 23 | 0.098 | 0.00021 | 0.041 | 0.095 | 1.7% |

**Table S4.** Rarefied and unrarefied normalisation comparison. The unrarefied dataset was normalised using a total-sum-scaled (TSS) transformation. R²- and *p*-value refer to the heatwave - water mass interaction term (100,000 permutations). PERMDISP homogeneity of multivariate dispersion test was performed among water masses.

| **Normalisation** | **Distance metric** | **R²-value  (interaction)** | ***p*-value  (interaction)** | **PERMDISP *p*-value** | |
| --- | --- | --- | --- | --- | --- |
| Rarefied (14,883 reads) | Bray-Curtis | 0.101 | 0.0061 | | 0.246 |
| Unrarefied (TSS) | Bray-Curtis | 0.102 | 0.0052 | | 0.231 |
| Rarefied (14,883 reads) | Hellinger-Euclidean | 0.09 | 0.018 | | 0.107 |
| Unrarefied (TSS) | Hellinger-Euclidean | 0.09 | 0.017 | | 0.097 |

2. **Dissimilarity metric sensitivity**

The primary PERMANOVA (Bray-Curtis) was repeated using two additional dissimilarity metrics representing different underlying assumptions: Hellinger-Euclidean distance (an abundance-weighted alternative standard in numerical ecology) and Jaccard distance (presence/absence, testing community turnover independent of relative abundance). The heatwave – water mass interaction remained significant under all three metrics (Table S5), with consistent effect sizes (R²= 0.090–0.101). Multivariate dispersion (PERMDISP) did not differ significantly by water mass under Bray-Curtis or Hellinger-Euclidean distances, indicating that the significant water mass PERMANOVA term under these metrics reflect genuine differences in community composition (a location effect) rather than differences in within-group variability (a dispersion effect). A significant dispersion effect was detected under Jaccard distance (*p* = 0.038). Follow-up analysis compared the coefficient of variation (CV = SD/mean) in pre-rarefaction sequencing depth across the three water masses (CV; STW = 0.31, Front = 0.07, SASW = 0.15) against their mean Jaccard dispersion (distance to group centroid: STW = 0.60, Front = 0.53, SASW = 0.55). Both measures ranked the water masses in the same order with STW the highest and Front the lowest. This is consistent with more variable sequencing depth being associated with greater apparent dispersion under a presence/absence-based metric. When testing this more directly by calculating the Spearman correlation between each of the 28 individual samples’ raw sequencing depth and its own distance-to-centroid value, no significance was reached (Spearman's ρ = −0.325, *p* = 0.092). This suggests depth-related variability may partly contribute to the Jaccard-specific dispersion pattern, though the evidence is suggestive rather than conclusive, and does not affect interpretation of the primary Bray-Curtis result, which showed no dispersion artifact (Table S5).

**Table S5.** Sensitivity of the primary PERMANOVA to dissimilarity metrics. All values are given at 100,000 permutations. PERMDISP *p*-value is reported for water mass only.

| **Distance metric** | **Term** | **R²-value** | ***p*-value** | **PERMDISP  *p*-value** | |
| --- | --- | --- | --- | --- | --- |
| Bray-Curtis | Heatwave | 0.184 | 0.00001 |  | |
| Bray-Curtis | Water mass | 0.180 | 0.00001 | | 0.246 |
| Bray-Curtis | Heatwave × Water mass | 0.101 | 0.00610 | |  |
| Hellinger-Euclidean | Heatwave | 0.166 | 0.00001 | |  |
| Hellinger-Euclidean | Water mass | 0.170 | 0.00002 | | 0.107 |
| Hellinger-Euclidean | Heatwave × Water mass | 0.090 | 0.01800 | |  |
| Jaccard | Heatwave | 0.099 | 0.00001 | |  |
| Jaccard | Water mass | 0.135 | 0.00003 | | 0.038 |
| Jaccard | Heatwave × Water mass | 0.092 | 0.00610 | |  |

3. **Jackknife analysis**

To assess whether the primary PERMANOVA result (significant heatwave – water mass interaction) depended on any individual sample, the analysis was repeated 28 times, each time excluding one sample. The heatwave – water mass interaction remained statistically significant (p < 0.05) under exclusion of every sample (Table S6). Most influential samples were drawn from the single MHW sampling campaign (P10). consistent with the reduced replication of the MHW samples relative to the non-MHW samples in this study design.

**Table S6.** Jackknife robustness of the primary PERMANOVA. The jackknife analysis is a leave one sample out approach. The 10 most influential samples out of the full 28 are shown. All values are given at 100,000 permutations.

| **Dropped sample** | **R²-value  (interaction)** | ***p*-value (interaction)** | **R²-value (MHW)** | ***p*-value (MHW)** | **R²-value  (water mass)** | ***p*-value (water mass)** |
| --- | --- | --- | --- | --- | --- | --- |
| (Full dataset) | 0.101 | 0.0061 | 0.184 | <0.0001 | 0.18 | <0.0001 |
| P10S8 | 0.083 | 0.0414 | 0.197 | <0.0001 | 0.172 | <0.0001 |
| P10S7 | 0.086 | 0.0311 | 0.197 | <0.0001 | 0.174 | <0.0001 |
| P10S1 | 0.094 | 0.0226 | 0.162 | <0.0001 | 0.182 | <0.0001 |
| P10S2 | 0.095 | 0.0188 | 0.162 | <0.0001 | 0.185 | <0.0001 |
| P14S2 | 0.100 | 0.0127 | 0.195 | <0.0001 | 0.167 | <0.0001 |
| P14S3 | 0.100 | 0.0123 | 0.195 | <0.0001 | 0.167 | <0.0001 |
| P10S6 | 0.100 | 0.0116 | 0.174 | <0.0001 | 0.183 | <0.0001 |
| P10S4 | 0.100 | 0.0108 | 0.175 | <0.0001 | 0.182 | <0.0001 |
| P10S3 | 0.100 | 0.0106 | 0.163 | <0.0001 | 0.186 | <0.0001 |
| P10S5 | 0.101 | 0.0102 | 0.170 | <0.0001 | 0.184 | <0.0001 |

4. **Variable selection stability (dbRDA)**

To assess the stability of environmental variable selection in the dbRDA, forward selection was repeated in a jackknife (leave-one-sample-out) design for 28 iterations and cross-checked against backward elimination on the full dataset. Temperature, chlorophyll-*a*, and phosphate, the variables retained in the final reported model, were selected in 100% of jackknife iterations and had the three lowest (most significant) individual removal p-values under backward elimination, despite no variable being formally excluded by that procedure. This provides convergent support for their identification as robust predictors of community composition.

**Table S7.** dbRDA environmental variable selection stability. Jackknife (leave-one-sample-out) forward selection repeated across 28 iterations (999 permutations per iteration); backward elimination performed once on the full dataset (100,000 permutations).

| **Variable** | **Selected in original forward selection** | **Jackknife selection frequency** | **Backward elimination removal  *p*-value** | **Retained under backward elimination** |
| --- | --- | --- | --- | --- |
| Temperature | Yes (1st) | 100% (28/28) | 0.0230 | Yes |
| Chlorophyll-*a* | Yes (2nd) | 100% (28/28) | 0.0004 | Yes |
| Phosphate | Yes (3rd) | 100% (28/28) | 0.0070 | Yes |
| CO_2_ | No | 61% (17/28) | 0.0490 | Yes |
| pH | No | 4% (1/28) | 0.0340 | Yes |
| Silicate | No | 11% (3/28) | 0.0560 | Yes |
| Salinity | No | 7% (2/28) | 0.0780 | Yes |

**Section D. Taxonomy**

Dinoflagellata

Chlorophyta

Ochrophyta

Ciliophora

Sagenista

Haptophyta

Prasinodermaphyta

Division

**Fig. S4:** Class-level taxonomical composition of protist communities in January 2017/2019/2020 (non-MHW), and in Januari 2018 (MHW). STW: Subtropical Water, Front: Southland Front, and SASW: sub-Antarctic Surface Water.

**Fig. S5:** Heatmap of the top most abundant ASVs in Subtropical water. ‘No MHW’ represents the summer cruises in January 2017, 2019, and 2020. MHW = marine heatwave, represents the January 2018 cruise. All individual samples are shown along the x-axis: *n* = 4 (No MHW) and *n* = 3 (MHW). The y-axis represents the Division level and ASV number with the lowest possible assigned taxonomic rank down to genus. ASVs identified to ‘Dinoflagellata| Dino-Group-I-Clade’ belong to the Syndiniales Class, while all other Dinoflagellata present here belong to the Dinophyceae Class. ASVs identified as ‘Chlorphyta| Chloroparvula’ belong to the Chloropicophyceae Class.

**Fig. S6:** Heatmap of the top most abundant ASVs in the Southland Front. ‘No MHW’ represents the summer cruises in January 2017, 2019, and 2020. MHW = marine heatwave, represents the January 2018 cruise. All individual samples are shown along the x-axis: *n* = 3 (No MHW) and *n* = 3 (MHW). The y-axis represents the Division level and | ASV number with the lowest possible assigned taxonomic rank down to genus. ASVs identified to ‘Dinoflagellata| Dino-Group-I-Clade’ belong to the Syndiniales Class, while all other Dinoflagellata present here belong to the Dinophyceae Class. ASVs identified as ‘Chlorphyta| Chloroparvula’ belong to the Chloropicophyceae Class.

**Fig. S7:** Heatmap of the top most abundant ASVs in sub-Antarctic surface water. ‘No MHW’ represents the summer cruises in January 2017, 2019, and 2020. MHW = marine heatwave, represents the January 2018 cruise. All individual samples are shown along the x-axis: *n* = 13 (No MHW) and *n* = 3 (MHW). The y-axis represents the Division level and | ASV number with the lowest possible assigned taxonomic rank down to genus. ASVs identified to ‘Dinoflagellata| Dino-Group-I-Clade’ belong to the Syndiniales Class, while all other Dinoflagellata present here belong to the Dinophyceae Class. ASVs identified as ‘Chlorphyta| Chloroparvula’ and ‘Chlorphyta| Chloropicon’ belong to the Chloropicophyceae Class.
